# The interplay between prolactin and cardiovascular disease

**DOI:** 10.3389/fendo.2022.1018090

**Published:** 2023-01-10

**Authors:** Andrea Glezer, Mariana Ramos Santana, Marcello D. Bronstein, Jose Donato, Raquel Soares Jallad

**Affiliations:** ^1^ Neuroendocrine Unit, Division of Endocrinology and Metabolism, Hospital das Clinicas, University of Sao Paulo Medical School, São Paulo, SP, Brazil; ^2^ Laboratory of Cellular and Molecular Endocrinology LIM-25, University of Sao Paulo Medical School, São Paulo, SP, Brazil; ^3^ Department of Physiology and Biophysics, Instituto de Ciencias Biomedicas, Universidade de Sao Paulo, Sao Paulo, SP, Brazil

**Keywords:** metabolic syndrome, hyperglycemia, systemic arterial hypertension, prolactinoma, cardiovascular risk, dyslipidemia, dopaminergic agonist, prolactin

## Abstract

Hyperprolactinemia can be caused by several conditions and its effects on the hypothalamic-pituitary-gonadal axis are understood in more detail. Nevertheless, in recent decades, other metabolic effects have been studied and data pointed to a potential increased cardiovascular disease (CVD) risk. A recent study showed a decrease in total and LDL- cholesterol only in men with prolactinoma treated with dopamine agonists (DA) supporting the previous results of a population study with increased CVD risk in men harboring prolactinoma. However, other population studies did not find a correlation between prolactin (PRL) levels and CVD risk or mortality. There is also data pointing to an increase in high-density lipoprotein levels, and decreases in triglycerides, carotid-intima-media thickness, C-reactive protein, and homocysteine levels in patients with prolactinoma on DA treatment. PRL was also implicated in endothelial dysfunction in pre and postmenopausal women. Withdrawal of DA resulted in negative changes in vascular parameters and an increase in plasma fibrinogen. It has been shown that PRL levels were positively correlated with blood pressure and inversely correlated with dilatation of the brachial artery and insulin sensitivity, increased homocysteine levels, and elevated D-dimer levels. Regarding possible mechanisms for the association between hyperprolactinemia and CVD risk, they include a possible direct effect of PRL, hypogonadism, and even effects of DA treatment, independently of changes in PRL levels. In conclusion, hyperprolactinemia seems to be associated with impaired endothelial function and DA treatment could improve CVD risk. More studies evaluating CVD risk in hyperprolactinemic patients are important to define a potential indication of treatment beyond hypogonadism.

## Introduction

Prolactin (PRL), as a classic hormone, is synthesized and secreted mainly by lactotroph cells from the anterior pituitary gland, which are tonically inhibited by hypothalamic dopamine (1). PRL is also secreted by extra-pituitary sources such as adipose tissue (2), with autocrine and paracrine actions, playing a dual role as a hormone and cytokine (2, 3). PRL can be classified according to its molecular weight into monomeric, dimeric, and macroprolactin, being monomeric the predominant isoform. Nevertheless, if the main isoform in circulation is macroprolactin, a condition known as macroprolactinemia can occur, without the clinical picture normally observed in hyperprolactinemia, as macroprolactin presents a low biological activity (4). Additionally, PRL can be cleavaged by proteases into vasoinhibin, with anti-angiogenic properties, being 16 kDa fragment related to peripartum cardiomyopathy (5).

The diagnosis of hyperprolactinemia is defined when a single measurement of serum PRL is above the upper limit of normal, if there was no excessive venipuncture stress (6) and there are several causes of hyperprolactinemia as physiological (pregnancy and lactation), pharmacological (especially antipsychotic drugs), primary hypothyroidism, renal failure, hepatic insufficiency, pituitary stalk disconnection and pituitary tumors with autonomous prolactin secretion as somatotrophinomas and prolactinomas (7). Pathological hyperprolactinemia can cause hypogonadotrophic hypogonadism, infertility, and galactorrhea (7), and the gold-standard treatment for prolactinoma is the use of dopaminergic agonists (DA) (7). Microprolactinomas in asymptomatic individuals, without bothersome galactorrhea, bone loss, and no fertility desire, could be followed without specific treatment (8). However, data associating hyperprolactinemia with other comorbidities such as obesity, metabolic syndrome, diabetes, and cardiovascular risk raise the question if serum PRL levels should be always normalized (9).

Cardiovascular disease (CVD) is the leading cause of death worldwide, and its global prevalence has increased every year (10, 11). There are traditional and established risk factors for CVD such as systemic arterial hypertension, dyslipidemia (12), atherosclerosis, insulin resistance (13), hyperglycemia (14), and obesity (15). Interestingly enough, there are literature data associating PRL with insulin resistance (16), hyperglycemia (17), and weight gain (18, 19), contributing indirectly to CVD risk. Also, there are case-controls, cohorts and populational studies evaluating PRL with CVD and the results are controversial.

This review focuses on the role of PRL and hyperprolactinemia on important CVD risk factors such as systemic arterial hypertension, dyslipidemia, atherosclerosis, endothelial dysfunction, glucose metabolism, and body weight. We also reviewed cohorts, case-control, and populational studies with normoprolactinemic individuals, and hyperprolactinemic patients, using or not DA, regarding CVD outcomes.

## Systemic arterial hypertension

PRL has biological effects on water and salt balance in different species (2, 20), including humans (21–23). In humans, two studies have shown that increased levels of PRL were associated with elevated arterial pressure in women with hypertension (24) and normotensive pregnant women (25). Recently, a cohort study has demonstrated that a higher daytime plasma PRL level, even within the normal range, was associated with an increased risk of incident hypertension among postmenopausal women from the Nurses’ Health Study (26). The levels of PRL in the urine were significantly higher in patients with preeclampsia than in subjects with normal pregnancy and antiangiogenic PRL fragments (14-16 kDa) in urine was detected only in patients with severe preeclampsia (27, 28). Cord blood PRL was high in newborns of hypertensive women (29).

Although in animal models, there were controversial results, in transgenic mice with inducible hepatic production of PRL and its cleavage product, an increase in PRL increased blood pressure by modulating the activity of endothelial nitric oxide synthase (30). Beyond PRL, dopamine also has a role in hypertension. Dysregulation of dopamine-dependent mechanisms has been pointed to as a determinant of hypertension, in studies in dopamine receptor knockout mice (31).

In a cross-sectional study, PRL levels within the normal range were associated with blood pressure values and arterial stiffness (32) but in another study, there were no differences in central, peripheral blood pressure, and arterial stiffness between patients with idiopathic hyperprolactinemia and controls (33).

## Dyslipidemia

Most studies have shown that patients harboring prolactinoma have modestly increased total cholesterol and LDL cholesterol levels (17, 34–39) with decreasing levels after dopamine agonist (DA) treatment and normalization of serum PRL levels (17, 38–47), which can occur independently of PRL levels (48). In some studies, cholesterol and LDL levels were decreased only in men (49). DA dose and length of treatment were variable, from 10 weeks (49) to 60 months (45, 48), differences that may interfere with the results found. Higher plasma triglycerides levels (17, 34, 50) and low HDL cholesterol (39, 50, 51) levels were also described in some cohorts of patients with prolactinoma, with decreasing levels of triglycerides after DA treatment in some studies (17, 46, 48).

**Figure 1:**
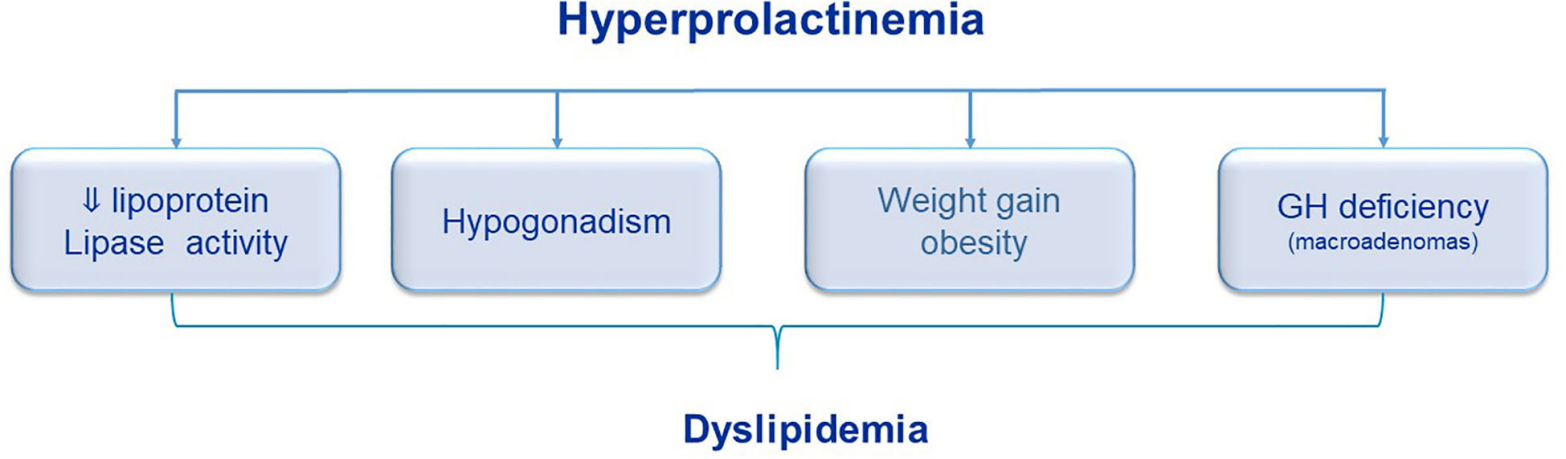
Potential role of hyperprolactinemia on dyslipidemia.

The mechanisms associating PRL with dyslipidemia are not completely understood and there are some hypotheses: 1) PRL decreases lipoprotein lipase activity in human adipose tissue and plasma lipoprotein lipase activity is decreased in patients with prolactinomas (34, 52); 2) hyperprolactinemia causes hypogonadism, which is a risk factor for hypercholesterolemia; 3) hyperprolactinemia can be related to weight gain and obesity, causing adverse lipid profile (17, 36) and 4) in macroprolactinomas with impairment of GH secretion, it could result in abnormal lipid levels (34) (**Figure 1**).Treatment with DA (bromocriptine or cabergoline) decreased LDL cholesterol and triglycerides levels in some studies and these effects could be related to the normalization of steroid sexual levels, and normalization of serum PRL levels, or, even, a direct effect of DA (53). In a study comparing metabolic outcomes in patients with prolactinoma treated by neurosurgery or DA, there was an improvement in lipid metabolism after a rapid decrease in PRL levels with surgery, while a high dose of CAB seemed to exert a beneficial impact on both insulin secretion and peripheral sensitivity (54). Auriemma et al (47) treated hypogonadic men with prolactinoma with CAB and testosterone replacement and showed that proper testosterone replacement induced a significant improvement in the metabolic profile, even though the amelioration in the lipid profile might reflect the direct action of CAB. Atorvastatin could reduce lipid levels in both hyperprolactinemic and normoprolactinemic women, with or without using DA (55).

As mentioned above, macroprolactinemia is usually a benign condition. However, compared with healthy controls, it was shown that women with isolated macroprolactinemia had increased levels of triglycerides and high sensitivity C-reactive protein (hsCRP), as well as lower levels of HDL cholesterol (56). Thereafter, the authors compared two groups of women with hypercholesterolemia treated with atorvastatin, one with macroprolactinemia and the other with normal PRL levels. The effect of atorvastatin in reducing cholesterol and LDL was more pronounced in the normoprolactinemic group (57).Comparing men with macroprolactinemia to subjects with monomeric hyperprolactinemia and normoprolactinemic men, the cardiometabolic risk was higher in macroprolactinemia compared to controls but to a lesser extent than in monomeric hyperprolactinemia (58).

## Atherosclerosis and endothelial dysfunction

*In vitro* studies have shown that PRL can modulate inflammatory responses, stimulate vascular smooth muscle cell proliferation, and play a role in the adhesion of circulating mononuclear cells to endothelium, pointing to a role of PRL in endothelial dysfunction (59). Reuwer et al. demonstrated that the PRL receptor was present in macrophages of the atherosclerotic plaque, proposing that prolactin receptor signaling contributes to the local inflammatory response within the atherosclerotic plaque and thus to atherogenesis (60).

PRL stimulates angiogenesis either directly, by promoting endothelial cell proliferation, or indirectly, by upregulating pro-angiogenic factors such as vascular endothelial growth factor (61). In addition, in experiments with animals, PRL might alter endothelial function through its vasoconstrictive features, reducing NO production directly (62), or indirectly, *via* its cleaved fragment 16KDa, contributing to an increase in blood pressure (30) and *via* its role in the trophic response of vascular smooth muscle (63).

An endothelial dysfunction is an event that precedes the formation of atherosclerosis and influences the progression of this disease and its adverse events. Endothelial function can be evaluated, *in vivo*, by measuring flow-mediated dilatation (FMD) on a brachial artery and/or measuring carotid-intima media thickness (CIMT). FMD was lower in patients in prolactinoma in some studies (50, 64, 65). In patients with prolactinomas, carotid-intima media thickness is increased when compared to healthy volunteers (50, 59, 66), with a decrease obtained after six months of DA treatment, independently of PRL levels (43). In one study including patients with prolactinoma treated with CAB who presented remission, at the moment of cessation of CAB therapy, the FMD percentage in patients with prolactinoma was worse than that in healthy controls and after the withdrawal of CAB treatment, fibrinogen, mean platelet volume, CIMIT, and HDL cholesterol were worse in the relapse patients than those in the remission patients. All these data pointed to a direct benefit of DA treatment in CVD risk (65).

Low-grade inflammation was also observed in hyperprolactinemic patients. High sensitivity C-reactive protein (hsCRP) was found in patients with prolactinoma (59, 64, 67) with decreasing levels after DA treatment (43, 64, 67). No differences in lipid profile and inflammatory markers were found in premenopausal women with hyperprolactinemia and hypercholesterolemia on atorvastatin, treated with BRC or metformin (68). After CAB treatment, inflammatory markers were reduced in two series of patients with prolactinoma (43, 67).

It is suggested that epicardial adipose tissue plays a role in adiposity-related inflammation and atherosclerosis through paracrine secretion of pro- and anti-inflammatory cytoquines (69). A recent study showed a greater thickness of epicardial adipose tissue in patients with prolactinoma, despite having normal systolic and diastolic cardiac functions (66).

Case-controls and retrospective/prospective studies evaluating dyslipidemia, atherosclerosis, and CVD risk factors in hyperprolactinemic patients are summarized in **Table 1** (**Table 1**). **Table 2** summarizes clinical trials, retrospective and prospective cohorts evaluating the effect of DA treatment in dyslipidemia, atherosclerosis, and CVD risk factors (**Table 2**).

**Table 1:** Studies with patients with hyperprolactinemia/prolactinoma without using DA.

Authors, year	Study design	Population studied	Results
Pelkonen et al, 1982 (34)	Case-control	Prolactinomas (N=44) *vs* healthy controls (N=8)	Cholesterol and TG higher in patients *vs* controls
Heshmati et al, 1987 (51)	Case-control	Women with prolactinomas (N=15) *vs* healthy matched-controls (N=15)	Cholesterol, LDL, and TG were similar in both groups, while was HDL was lower in the hyperprolactinemic group.
Oppenheim et al, 1989 (36)	Case-control	Men with prolactinoma and hypogonadism (N=18), men with secondary hypogonadism and normal PRL (N=15) *vs* healthy men in control (N=33)	Cholesterol, LDL, and TG are higher in patients with hypogonadism, with or without hyperprolactinemia
Erem et al, 2010 (35)	Case-control	Prolactinoma (N=22) *vs* healthy controls (N=20)	Increased levels of total cholesterol, LDL-cholesterol, apolipoprotein B, platelet count, fibrinogen, AT-III, PAI-1, and PAI-1/t-PA ratio in patients vs controls
Jiang et al, 2014 (50)	Case-control	Patients with prolactinoma without previous treatment (N=31) *vs* healthy patients (N=60)	Higher levels of triglycerides, ApoB/ApoA-1, CRP, and fibrinogen and lower HDL and ApoA-1 in patients *vs* controls Higher levels of PRL determined lower FMD of the brachial artery and higher carotid intimal mean thickness independent of traditional risk factors
Arslan et al, 2014 (59)	Case-control	Prolactinomas (N=35) *vs* healthy controls (N=36)	hs-CRP level and carotid intimal mean thickness were significantly higher in patients *vs* controls
Peric et al, 2016 (37)	Case-control	Patients with prolactinomas (N=29) *vs* clinically non-functioning pituitary adenomas (N=57)	Prolactinomas were associated with higher LDL cholesterol, DHEA-S, and lower GH levels
Soto-Pedre et al, 2017 (70)	Retrospective cohort	1204 individuals, followed for 10 years, divided into 4 groups: 1) Pituitary disorders (N=331): MIC (N=196), MAC (N=54), and without MRI reports (N=81) 2)Drug-induced hyperprolactinemia (N=598) 3)Hyperprolactinemia due to hypothyroidism (N=79) 4)Idiopathic hyperprolactinemia (N=196)	No increase in outcomes (death, diabetes, bone fractures, non-fatal cardiovascular disease, cancer, autoimmune disease, and infectious disease) was observed in patients with MIC. MAC carriers, drug-induced and idiopathic hyperprolactinemia groups with an increased risk of death. The increased risk was not related to PRL levels.
Toulis et al, 2018 (71)	Retrospective Cohort	Patients with prolactinoma (1822 women and 411 men)	Increased incidence of cardiovascular outcomes only in men
Koca et al., 2021 (33)	Case-control	Idiopathic hyperprolactinemia (N=54) vs healthy controls (N=55)	There were no differences in central, peripheral pressure, and arterial stiffness between groups or with PRL levels

MIC, microprolactinoma; MAC, macroprolactinoma; CAB, cabergoline; BRC, bromocriptine; DA, dopamine agonist; CRP, C reactive protein; PRL, prolactin; OR, odds ratio; CKI, chronic kidney insufficiency; T2DM, type 2 diabetes mellitus; FMD, flow mediated dilation; TG, triglycerides; AT-III, Antithrombin- III; PAI-1, plasminogen activator inhibitor 1; MRI, magnetic resonance imaging.

**Table 2:** Studies with patients with hyperprolactinemia/prolactinoma on DA treatment.

Authors, year	Study design	Population studied	Results
Fahy et al, 1999 (40)	Case-control	Women with hyperprolactinemia on BRC (N=15) *vs* healthy control (N=15)	No differences in the lipoprotein profile between cases and controls. On BRC, total cholesterol and LDL-cholesterol reduced
Yavuz et al, 2003 (64)	Case-control	Premenopausal women with prolactinomas on BRC (N=16) *vs* healthy controls (N=20)	Higher levels of Homocysteine, CRP, and acid uric and lower FMD of a brachial artery in cases, with improvement in BRC
Serri et al, 2006 (67)	Clinic Trial	Patients with hyperprolactinemia on CAB for 12 weeks (N=15)	After CAB, inflammatory markers reduced
Berinder et al, 2011 (38)	Clinic Trial	Prolactinomas (8 women, 1 postmenopausal) on BRC or CAB (N=14)	LDL reduced after 2 months on DA
dos Santos Silva et al, 2011 (46)	Prospective cohort	Patients with prolactinoma on DA (N=22)	Improvement of LDL-cholesterol and TG levels after 6 months on DA
Ciresi et al, 2013 (48)	Retrospective Cohort	Patients with prolactinoma on CAB (N=43)	On CAB, after 12 months, total and LDL- cholesterol, and TG were reduced and HDL-cholesterol levels increased, regardless of PRL levels
Inancli et al, 2013 (43)	Retrospective study	Women with prolactinoma on CAB (N=21)	Improvement in inflammatory markers and a decrease in carotid intimal mean thickness on CAB, after 6 months, regardless of PRL, LDL- cholesterol levels and BMI
Auriemma et al, 2013 (45)	Prospective study	Patients with prolactinomas on CAB (N=61)	Lipid profile improved after 12 and 60 months on DA
Krysiak et al, 2015 (39)	Clinical Trial	20 women with hyperprolactinemia: Group 1 = BRC-resistant on CAB for 6 months (N=8) Group 2 = hyperprolactinemia prolactinoma-non-related on BRC for 6 months (N=12)	Only CAB reduced triglycerides, CRP, homocysteine, fibrinogen, and increased cholesterol HDL. CAB was superior to BRC in the effect on free fatty acids, CRP, homocysteine, and fibrinogen.
Krysiak et al, 2015 (68)	Clinical Trial	Premenopausal women with hyperprolactinemia and isolated hypercholesterolemia (N=31) on atorvastatin 20mg for 12 weeks: Group 1 = on BRC (N=14) Group 2 =on metformin (N=17)	No differences in lipid profile and inflammatory markers were found after intervention in both groups
Pala et al, 2015 (17)	Case-control	Prolactinomas (N=19) evaluated metabolically at baseline, 3 and 6 months after on CAB *vs* healthy controls (N=20)	Increased levels of LDL-cholesterol and TG at baseline in patients *vs* controls. After 6 months on CAB, total cholesterol, LDL-cholesterol, and TG reduced
Medic-Stojanoska et al, 2015 (42)	Case-control	Premenopausal women with prolactinoma on DA (N=22) *vs* healthy controls (N=16)	Total cholesterol and LDL levels reduced on DA
Auriemma et al, 2015 (47)	Prospective study	Men with prolactinomas (N=32): with (N=22) or without (N=10) hypogonadism on CAB	Lipid parameters improved after 12 months on CAB with further ameliorating after 24 months
Dogan et al, 2016 (65)	Case-control	Premenopausal women, non-obese with MIC on CAB, evaluated at baseline, 3 months, and 12 months after CAB is withdrawn *vs* healthy controls (N=30)	FMD of the brachial artery was worse at baseline in patients *vs* controls Fibrinogen, mean platelet volume, mean carotid thickness, and HDL were worse in patients with relapses than in those with remission, after CAB is withdrawn. Only mean platelet volume was associated with recurrence of hyperprolactinemia
Schwetz et al, 2017 (41)	Clinical Trial	Patients with prolactinoma (N=53), 22 MIC, and 31 MAC, on CAB for 9 months until PRL normalization	Decrease in LDL levels after PRL normalization with DA
Krysiac et al, 2019 (55)	Clinical Trial	59 overweight premenopausal women with hypercholesterolemia divided into 3 groups, on atorvastatin for 12 weeks: Group A = hyperprolactinemia (N=19) Group B = normoprolactinemia on BRC (N=20) Group C = normoprolactinemia without DA (N=20)	Atorvastatin reduced lipid levels in all groups. Higher levels of acid uric, CRP, homocysteine, and fibrinogen in Group A
Khalil et al, 2021 (44)	Prospective observational	Patients with prolactinoma on BRC (N=32)	LD-cholesterol reduced on BRC
Yazici et al, 2021 (66)	Case-control	Patients with prolactinoma with and without CAB (N=67) *vs* healthy controls (N=57)	The greater thickness of epicardial adipose tissue and carotid intimal media in patients *vs* controls
Posawetz et al, 2021 (49)	Case-control	Patients with prolactinoma (MIC and MAC) with CAB in a 10-week- follow-up (N=21) *vs* healthy controls (N=30)	Reduction of total and LDL cholesterol with CAB in men with MAC
Pirchio et al, 2021 (54)	Clinical Trial	Patients with prolactinoma resistant to CAB on conventional doses (N=34): Group 1 (N=17): pituitary surgery. Group 2 (N=17): on CAB (>2mg/week)	The rapid decrease in PRL levels induced by pituitary surgery might improve lipid metabolism. No impact in medical therapy with high dose CAB.

MIC, microprolactinoma; MAC, macroprolactinoma; CAB, cabergoline; BRC, bromocriptine; DA, dopamine agonist; CRP, C reactive protein; PRL, prolactin; OR, odds ratio; CKI, chronic kidney insufficiency; T2DM, type 2 diabetes mellitus; FMD, flow mediated dilation; TG, triglycerides; AT-III, Antithrombin- III; PAI-1, plasminogen activator inhibitor 1; MRI, magnetic resonance imaging.

## Insulin resistance and hyperglycemia

PRL plays a role in glucose metabolism during pregnancy. Results from animal studies showed that PRL stimulates the growth of pancreatic islets, and insulin secretion during the perinatal period in the fetus (72, 73). In the mothers, the expression of the PRL receptor in the pancreatic islets increases during pregnancy (74), and the threshold for glucose-stimulated insulin secretion is reduced (73). However, two studies have shown PRL that levels during pregnancy were associated with gestational diabetes mellitus risk (75) and reduced tolerance glucose during pregnancy (76). Polymorphisms in the PRL receptor were associated with gestational diabetes (77). However, other studies have demonstrated an opposite result: an inverse association between PRL and risk for gestational diabetes mellitus (78, 79). Interestingly enough, lactation has been reported to significantly reduce the risk of metabolic syndrome, and type 2 diabetes (80). In a prospective cohort of women who presented gestational diabetes, decreasing prolactin quartiles in postpartum, associated with higher lactation intensity and duration, were also associated with increased future T2D risk and in women who maintained normoglycemia during the 10-year follow-up, higher prolactin at baseline was associated with higher insulin sensitivity (81).

In non-pregnant status, PRL also seems to play a role in insulin secretion, β-cell proliferation, and glucose metabolism (82–84). Data regarding PRL and glucose metabolism are controversial. In the normoprolactinemic population in general, higher PRL levels within the normal range were associated with improved insulin sensitivity and glucose metabolism, and lower prevalence of diabetes and metabolic syndrome (85–88). Nevertheless, some studies showed that PRL levels were inversely associated with T2D risk (89, 90), particularly in women (91, 92), while others show a positive or no association in clinical cohorts including men and women (93, 94). A recent meta-analysis (95) reported that a higher PRL level within the normal range was associated with reduced risk of prevalence but not incidence T2D.

**Figure 2:**
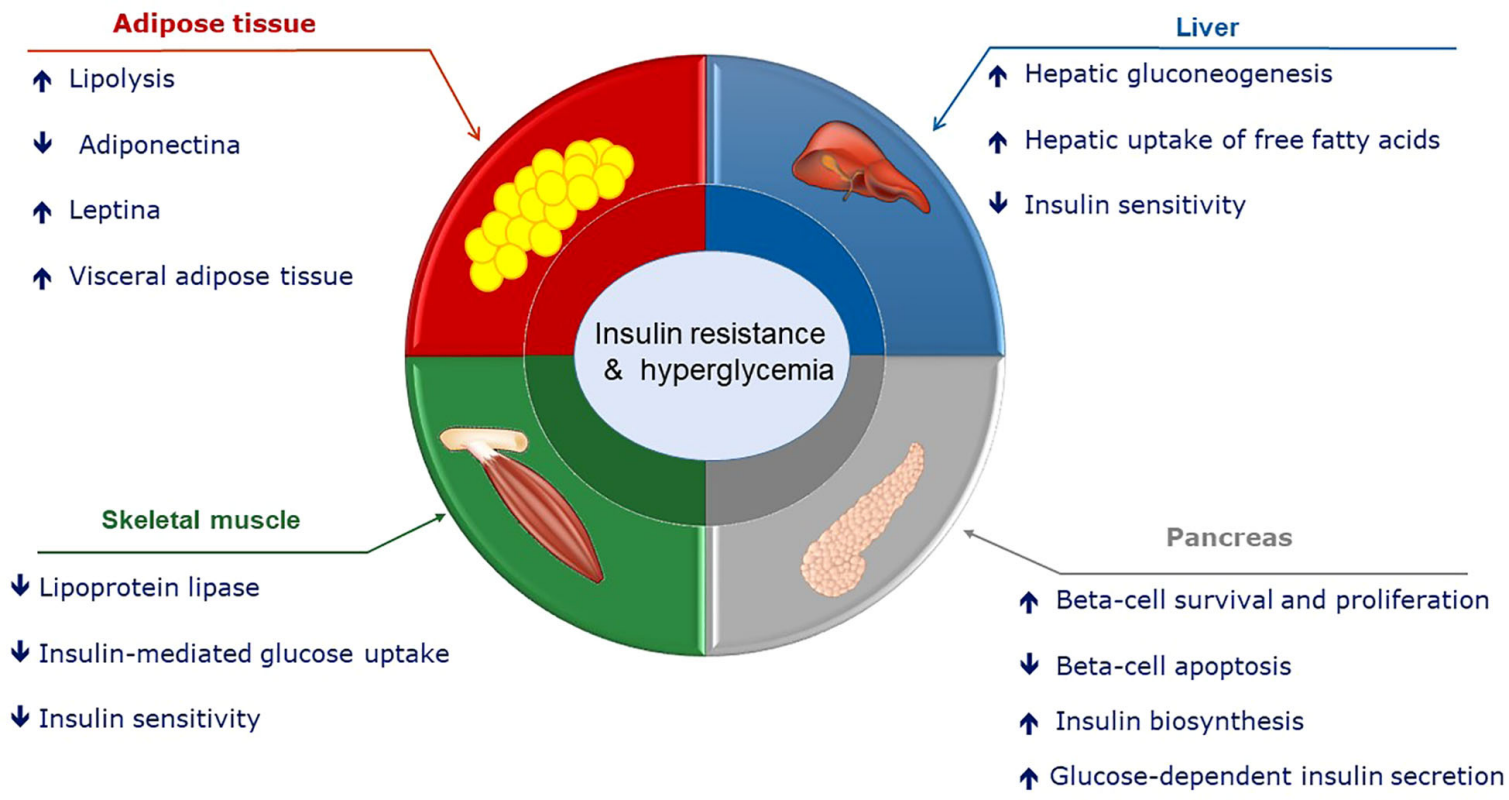
Potential role of hyperprolactinemia on glucose metabolism.

Regarding hyperprolactinemia in non-pregnant status, a study in 1977 showed a decrease in glucose tolerance and hyperinsulinemia, so the authors suggested a diabetogenic effect of PRL in hyperprolactinemic patients (96) (**Figure 2**). Using the hyperinsulinemic-euglycemic clamp technique, it has been shown that hyperprolactinemic patients were more insulin resistant than control subjects, and these findings were not associated with obesity or anthropometric parameters such as fat content, waist circumference, and body mass index (BMI) (97).

DA treatment seems to improve glucose metabolism alterations found in hyperprolactinemia. Hyperprolactinemia induced by hCG in female mice leads to metabolic disturbances such as hyperinsulinemia, hypertriglyceridemia, dyslipidemia, and glucose intolerance, and those disturbances were prevented by treatment with CAB (98). Dos Santos Silva et al. evaluated 22 patients with prolactinoma and after six months of treatment with DA and normal PRL levels, there was a significant decrease in homeostasis model assessment of insulin resistance (HOMA(IR)) index, normalization of glycemia, although no significant difference in BMI was observed (46). In another study including 43 patients with prolactinoma treated with CAB for 12 months, fasting insulin, HbA1c, and HOMA-IR were reduced, independently of PRL levels, while lower BMI was observed only in patients receiving higher doses (>0·50 mg/week) of CAB (48). Metabolic syndrome prevalence was higher in 61 patients with prolactinoma at baseline, especially with higher levels of serum PRL. Fasting insulin and HOMA(IR) significantly decreased after 1 year of CAB and further improved after 60 months (45).

The observed dose-dependent effect of PRL on glucose metabolism may provide a possible answer to understanding the conflicting data regarding PRL effects on glycemia. Increased insulin sensitivity in liver tissue as well as β-cell growth, leads to greater insulin production in the presence of slightly increased prolactin levels (99, 100). Moreover, it is also possible that mildly higher PRL levels may affect glucose metabolism in an indirect pathway *via* increasing dopamine secretion (100) (101). Low PRL levels were associated with higher risk of MS, polycystic ovary syndrome, postpartum diabetes and diabetes mellitus type 2 in population studies. In a recent study, prolactin receptor knock out mice fed with a high fat diet presented obesity, exacerbated glucose intolerance, insulin resistance, and enlarged adipocytes, compared to the wild type (87). However, more data supporting a causal role of low PRL levels to those findings are needed (102). PRL levels ~40 mcg/L were associated with lower prevalence of metabolic disease while PRL levels higher than 100 mcg/L were associated with deleterious metabolic alterations (91, 103). Therefore, some authors hypothesized that upregulation of PRL levels is a mechanism to maintain metabolic homeostasis and proposed that either very low levels of PRL (< 7 ng/mL) or higher levels of PRL (> 100 ng/mL) were associated with insulin resistance and metabolic syndrome. They suggest defining PRL levels in the range between 25 to 100 mcg/L with no other causal explanations as “HomeoFIT-PRL”, which could represent a physiological response to an increase in metabolic demand (102).

There is evidence that the phases and amplitudes of the plasma rhythms of prolactin and corticosterone are different between insulin-sensitive and insulin-resistant rats. Daily injections of corticosterone and prolactin to simulate the endogenous rhythms of these hormones observed in metabolically healthy animals improve insulin sensitivity and reduce body fat stores of insulin resistance, obese animals (104). Thus, the circadian rhythm of plasma prolactin plays an important role in determining the metabolic effects of this hormone on different tissues.Nothe

It is also important to stress that DA treatment may have a central effect on improving glucose metabolism, probably by regulating central nervous system dopaminergic activity. Thus, dysfunctions in prolactin release (either by hyperprolactinemia or low prolactin secretion) can dysregulate the circadian rhythm of central dopaminergic activity, especially at the level of the hypothalamic suprachiasmatic nucleus that works as our biological clock, leading to alterations in the systemic metabolism (105). This dopaminergic-clock regulatory neurocircuit modulates the activity of the sympathetic nervous system, affecting multiple metabolic processes like hepatic glucose production, adipose tissue lipolysis, and peripheral insulin sensitivity (106). It is also possible that a decreased central dopaminergic tone may alter the activity of the hypothalamic-pituitary-adrenal axis, which plays a fundamental role in controlling glucose homeostasis, body adiposity, and other metabolic aspects (107). A bromocriptine quick-release formulation was designed as a glucose-lowering drug and approved for clinical use by the FDA in 2009 (commercialized as CYCLOSET^®^). Recent evidence indicates that circadian-timed bromocriptine quick-release treatment reduces the sympathetic tone and improves systemic low-grade inflammation in type 2 diabetes subjects (108). Thus, these findings help to explain how circadian-timed DA administration is able to improve the metabolic status of patients and reduce the risk of cardiovascular diseases, independently of their effects on prolactin secretion. However, additional studies are necessary to investigate the potential metabolic effects of circadian-timed administration of DA in hyperprolactinemic subjects.

Finally, human and animal studies indicate that reduction in striatal dopamine causes a decreased peripheral insulin sensitivity in healthy subjects (109, 110). On the other hand, deep brain stimulation that results in dopamine release in the striatal area increases peripheral insulin sensitivity in diabetic and nondiabetic patients. Furthermore, optogenetic activation of dopamine D1 receptor-expressing neurons in the ventral striatum increases glucose tolerance and insulin sensitivity in mice (109). Thus, the central dopamine system regulates systemic glucose homeostasis, so this mechanism should be taken into account for the metabolic effects induced by DA treatment.

## Weight gain and obesity

Body weight is regulated by a complex system that coordinates food intake and energy expenditure. The neuroendocrine system involved in the control of energy balance and body composition comprises the action of several organs including the gastrointestinal tract (pancreas, small intestine, liver), brain, adrenals, and adipose tissue. PRL mRNA is expressed in all of these organs and PRL integrates endogenous or environmental signals to ensure metabolic homeostasis (111–113).

**Figure 3:**
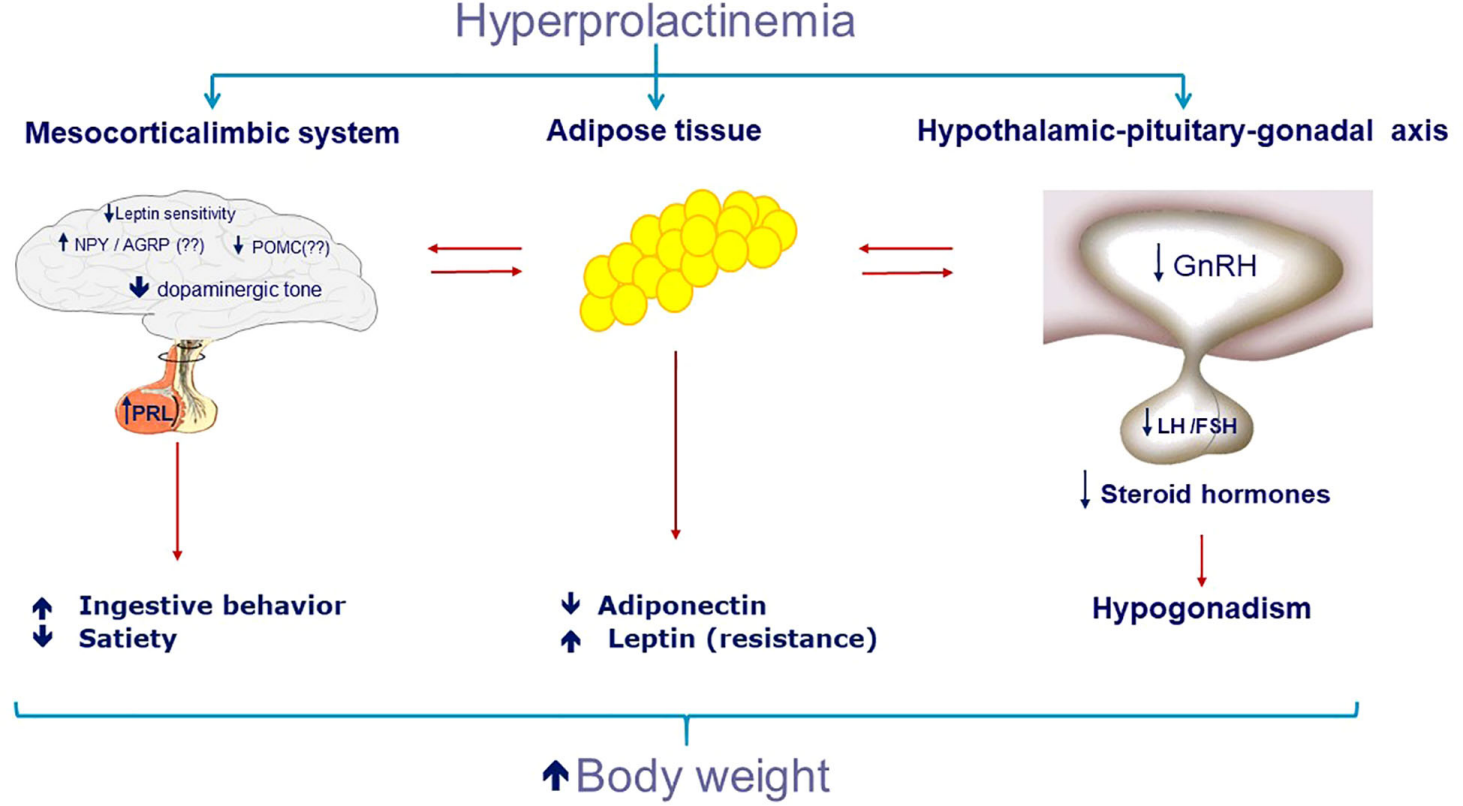
Potential role of hyperprolactinemia on increase of body weight.

Several animal and human studies (especially in men) describe a higher body weight in patients with hyperprolactinemia (67, 113–115). In some series, the prevalence of obesity and overweight reaches values ​​of 45 and 37%, respectively (46). The exact mechanism has not yet been fully elucidated and may include decreased dopaminergic tonus (101, 116), hypogonadism, low adiponectin (111, 117, 118), and leptin resistance (119)(**Figure 3**).

Central dopamine is a neurotransmitter, essential to the regulation of food intake, and serves to drive behaviors necessary for food consumption (120). In particular, the mesolimbic dopamine pathway in the midbrain serves to drive behaviors necessary for food consumption by integrating homeostatic signals and modulating the rewarding and motivational value of food (121, 122). Dopaminergic neurons project from the ventral tegmental area (VTA) to the nucleus accumbens and participate in the regulation of the brain reward pathway (123, 124). Several studies point to the role of dopamine in the development and maintenance of diet-induced obesity (125). Also observed in obesity, chronic low-grade inflammation can disturb dopamine tone, which can result in the inability to put in the effort required to achieve the desired goal (123). The two dopamine receptors classes D1 receptor (D1R and D5R) and D2 receptor (D2R, D3R and D4R) are present in the cerebral cortex, the limbic system and the striatum (126). The D1R can influence appetite motivation, stimulating the individual to eat more food. The D2R is also expressed in the pituitary and in the hypothalamus, and is associated with saciety (124, 126, 127). Molecular imaging studies showed structural dopamine alterations in human obesity, especially lower dopamine D2/D3 receptor expression in striatal regions in extreme obesity (128, 129). In contrast to Wang and colleagues (130), most studies show no linear correlation between BMI and D2/D3 receptor binding potential (131).For mild to severe obesity, the evidence points towards higher striatal D2/D3 receptor binding potential, although the picture is less clear (132). Animal studies have found that insulin interacts with dopamine in the VTA and striatum (133) and the administration of insulin directly into the VTA suppresses ingestive behavior (133). Dopaminergic tone suppression has been considered as the mechanism contributing to increased food intake and weight gain in hyperprolactinemia obesity (18, 101, 134–136), together with increased hypothalamic levels of the appetite-stimulating hormones neuropeptide Y and corticotrophin-releasing hormone (136–138). Treatment with DA in hyperprolactinemic patients can lead to weight reduction possibly by increasing dopaminergic tone, beyond normalization of PRL levels and reverting hypogonadism (138).

Regarding sex differences, men with hyperprolactinemia presented higher risk of cardiovascular disease (71) and higher mortality rates (139). Only men with prolactinoma presented significantly higher fat mass and higher levels of cholesterol and LDL-cholesterol, compared to controls (49). On the other hand, in patients harboring prolactinoma, weight and body mass index reduction were more pronounced in men than women, after six months of bromocriptine treatment (101). Usually, men with prolactinoma are later diagnosed compared to women, being exposed to hyperprolactinemia and hypogonadism for a longer period. Additionally, men presented macroprolactinomas in a higher frequency, what could be related to a longer time to recover gonadal axis after treatment is initiated. These aspects could differently impact cardiovascular risks in both sexes.

**Figure 4:**
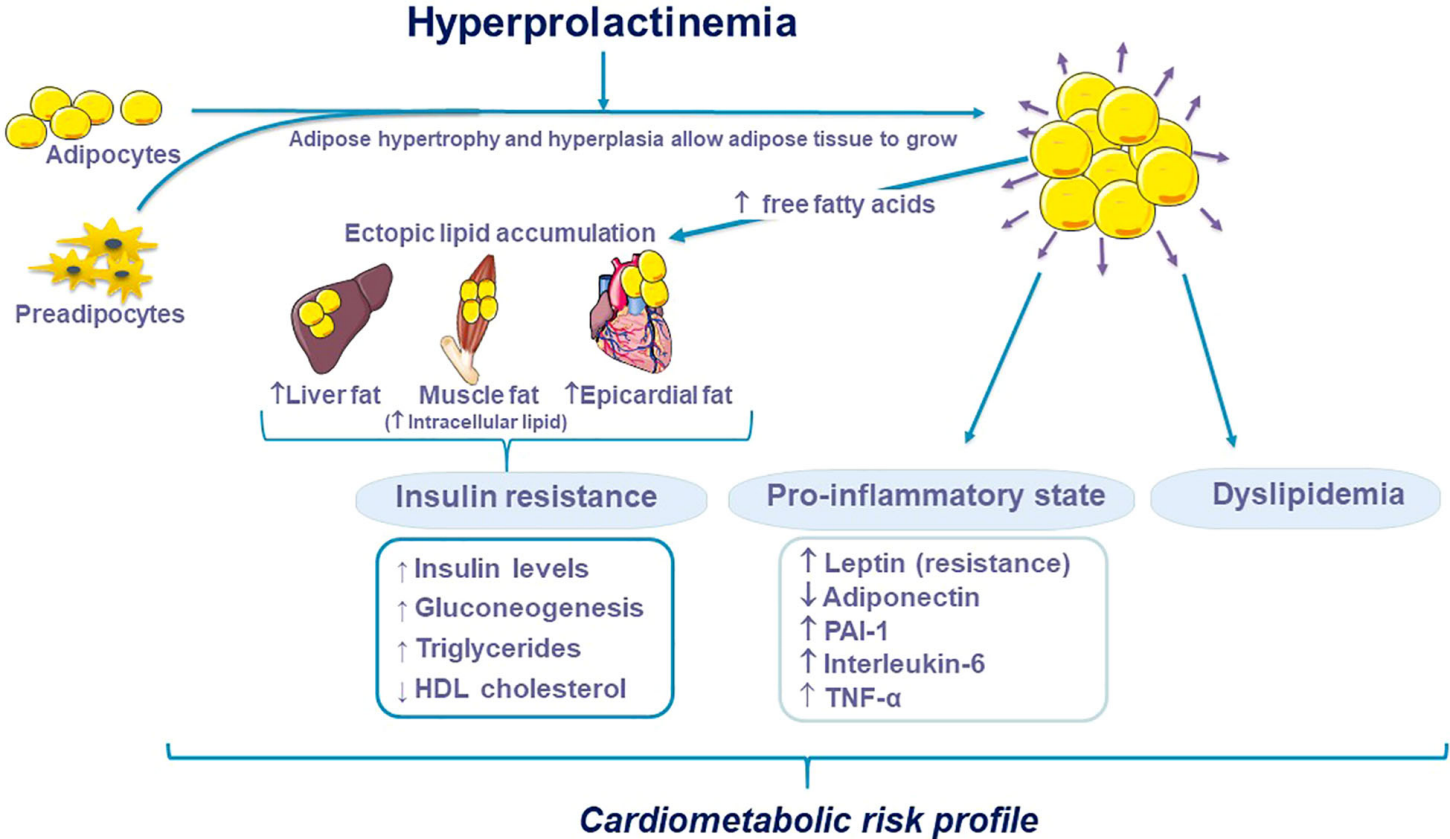
Potential role of hyperprolactinemia on adipose tissue.

Adipocytes and preadipocytes possess both androgen (140) and estrogen (141) receptors and an increase in body fat has been demonstrated in hypogonadal women. Then, hyperprolactinemia-induced hypogonadism may explain the changes in the amount of body fat. However, although patients with hypogonadism have higher median levels of PRL, it was not possible to demonstrate weight differences according to gonadal function. PRL has been associated with changes in adiposity and appears to participate in multiple aspects of adipose tissue biology, including adipogenesis, lipolysis, and the release of adipokines such as adiponectin or leptin (121). During adipogenesis, there is an increase in the expression of PRL receptors, which promote adipogenesis through an increase in essential transcription factors such as the peroxisome proliferator-activated receptor-γ (142). In the study by Auffret et al., the absence of PRL signaling could have induced the formation of brown adipocytes, favoring the hypothesis that PRL may be involved in the differentiation of beige adipose tissue from white adipose tissue (143). On the other hand, some authors showed that, in mice, pathological hyperprolactinemia has a strong impact on brown adipose tissue, lowering thermogenic markers and evoking tissue whitening (111) (**Figure 4**).

Although PRL decreases serum levels of adiponectin, by influencing its expression and secretion *in vitro*, in patients with prolactinomas there was no association between PRL and adiponectin levels (38). Regarding leptin, its synthesis and secretion are increased either through a direct action of PRL or indirectly through the increase of pro-inflammatory cytokines (121, 144). In a cohort of patients with prolactinoma, treatment with DA led to a reduction in BMI, and leptin levels were associated with BMI but not with serum PRL (101).

Human adipose tissue is a site of prolactin synthesis and secretion (145, 146). Adipose tissue is surrounded by connective tissue with proteoglycans rich in heparin sulfate, to which prolactin binds, causing most of the locally produced prolactin to be retained (147). It can be speculated that, when produced in large amounts, some of the PRL reaches the peripheral circulation, while when produced in smaller amounts it is retained by the producing cells, transforming prolactin into a true paracrine or autocrine agent. Corroborating this hypothesis, a study showed an increase in basal and pulsatile prolactin secretion in premenopausal women with visceral obesity, compared to lean women (146).

In obese humans, PRL levels were increased in metabolically healthy obesity. The authors suggested that increased circulating PRL might be a compensatory response for favoring energy metabolism during obesity (148).

Hyperprolactinemia is commonly associated with weight gain and obesity in humans (18, 101), although some findings are contradictory (67, 149). Animal studies indicate that PRL acts on the brain by stimulating food consumption, increasing body fat deposits, and promoting resistance to leptin and insulin (150–154). Hyperprolactinemia induces functional blockade of dopaminergic tone, which can be considered among the factors involved in the pathogenesis of hyperphagia and weight gain observed in patients with hyperprolactinemia, thus contributing to obesity.

The waist circumference and BMI of patients with prolactinomas were found to be significantly higher than in the controls (155). While body fat percentage was similar in nonobese women with prolactinoma compared to controls (19), newly-diagnosed men with prolactinomas had higher body fat content (156). Greenman et al. showed that in patients with prolactinoma treated with DA there was weight reduction in 70% of patients and 90% of men with normal PRL levels (18). Similar results were found in two other studies (101, 157), one of them with more pronounced results in men (101). Despite the high prevalence of obesity and overweight in a cohort of patients with prolactinoma, six months of DA treatment and normal PRL levels did not lead to a significant difference in BMI (46). In other studies, with longer treatment duration, the visceral adiposity index significantly decreased after CAB treatment: 12 months, regardless of the degree of reduction in PRL levels (48), 60-months CAB treatment compared to baseline (45). In a recent systematic review and meta-analysis evaluating metabolic effects of DA in patients with prolactinomas, the pooled standardized mean difference of the primary outcome revealed a reduction in BMI and weight and a subgroup analysis suggested that the reduction of weight was primarily driven by studies with high prolactin levels at baseline (158).

Then, *in vivo* and *in vitro* work has demonstrated that prolactin may increase or decrease adipogenesis. PRL in physiological concentrations plays a role in adipogenesis, adipocyte differentiation, and protection from metabolic syndrome, while in physiological hyperprolactinemia during pregnancy and lactation it presents lipogenic activity. In pathological hyperprolactinemia, PRL predisposes to obesity, induces visceral fat depot hypertrophy, and decreases lipogenesis (112).

## Peripartum cardiomyopathy

Another interesting cardiovascular effect described in association with hyperprolactinemia is peripartum cardiomyopathy (PPCM) (159). This is a rare disease associated with late pregnancy or the peripartum period, marked by severe systolic dysfunction leading to reduced ejection fraction and symptoms of heart failure (159, 160). It has been shown that in these patients, for reasons still unknown, cleavage of PRL from its 23 kDa form to a 16 kDa form by cathepsin-D occurs (160). This 16 kDa PRL induces endothelial cell apoptosis, vasoconstriction, reduced metabolism, and cardiomyocyte function, leading to PPCM (161). In 2010, a pilot study using standard treatment associated with bromocriptine in women with PPCM showed an improvement in ejection fraction at 6 months when compared to the standard therapy group (162). A recent multi-centre randomized study showed that in severe PPCM, patients observed a high left ventricular recovery rate at 6 months with no mortality, even with short-term treatment with bromocriptine (2.5 mg daily for one week) (163) and in 2018 ESC (European Society of Cardiology) Guidelines for the management of cardiovascular diseases during pregnancy states that bromocriptine may be considered in women with newly diagnosed PPCM (164).

## Populational studies

In an observational study including 457 normoprolactinemic non-dialyzed CKD patients and 173 hemodialysis patients, PRL levels were directly associated with endothelial dysfunction/stiffness and with increased risk of cardiovascular events and mortality (165). Then, a positive association between serum PRL concentrations and all-cause mortality and cardiovascular mortality was first reported in 2014, with a significant trend across PRL tertials, in a population-based Study of Health in Pomerania, including 3929 individuals (1946 men and 1983 women) aged 20–81 (mean 50.3 years) (166). Dourado et al. reviewed the main common aspects of CKD, prolactinemia, and cardiovascular risk (167). In a study conducted in Denmark, from a cohort of 3633 patients with a median follow-up time of 5.3 years, mean age of 39.7 years, and 78% of females, 10.3% presented hyperprolactinemia, without a history of pituitary disease. In males, hyperprolactinemia was associated with a high age-adjusted incidence ratio for cardiovascular mortality, even after adjusting for confounders such as chronic renal failure, diabetes, and antipsychotic medication (139). Finally, a population-based, retrospective, open-cohort study in the UK included 2233 patients with prolactinoma and 10355 age, sex, BMI, and smoking status-matched controls (1:5 ratio) demonstrated that the incidence of CVD was higher in the prolactinoma group, only amongst males, even after covariate adjustment (71). In a retrospective study including 1204 individuals, followed for 10 years, divided into 4 groups: pituitary disorders (N=331), drug-induced hyperprolactinemia (N=598), hyperprolactinemia due to hypothyroidism (N=79), and idiopathic hyperprolactinemia (N=196), there was an increased risk of death in patients harboring macroadenomas, drug-induced and idiopathic hyperprolactinemia groups, although not related to PRL levels.

However, in other studies, PRL levels were inversely correlated to the risk of diabetes and dyslipidemia. In a large population-based study including 2,377 participants (1,034 men and 1,343 postmenopausal women) without hyperprolactinemia, aged 40 years and older, in Shanghai, China (85), low PRL levels were associated with risk of diabetes. In another study including 8615 women from the NHS and NHSII, with documented normal serum PRL levels, divided into quartiles, followed up to 10 years, there were 99 incident type 2 diabetes cases reported and diabetes incidence was correlated with the lowest quartile (91), irrespective to menopausal status. In an Indian case-control study, PRL in T2DM patients (n=112) was significantly lower compared to healthy controls (n=112) and PRL levels were inversely correlated with total cholesterol, LDL-cholesterol, and triglycerides (70).

Moreover, PRL levels were not associated with CVD in the other two populational studies. No association was found between normal PRL levels and cardiovascular risk factors in a study including 3232 individuals, mean age 40.4 years, 52.1% women, from the Framingham Heart Study participants who attended 2 examinations an average of 6.1 years apart (94). In a case-control study from a prospective EPIC-Norfolk cohort, men and women, aged 45 to 79 years, who developed fatal or nonfatal coronary arterial disease (882) were compared to controls (1490) regarding serum PRL levels and there was no association between the highest prolactin tertile and CVD (168).

Studies in normoprolactinemic individuals evaluating dyslipidemia, CVD, and CVD mortality populational studies evaluating PRL, CVD risk factors, and CVD mortality are summarized in tables (**Table 3**) (**Table 4**).

**Table 3:** Studies with normoprolactinemic individuals.

Authors, year	Study design	Population studied	Results
Georgiopoulos et al, 2009 (32)	Cross-sectional study	Postmenopausal women (N=76)	PRL levels associated with blood pressure and arterial stiffness
Reuwer et al, 2009 (168)	Prospective Cohort	Patients with fatal or nonfatal CVD (N=882) and healthy controls (N=1490)	No association between higher tertile levels of PRL and CVD
Carrero et al, 2012 (165)	Prospective cohort	Patients with CKI without dialysis (N=457) and 173 in dialysis (N=173).	PRL is associated with endothelial dysfunction/stiffness and with an increased risk of CVD and mortality in both groups
Jayashankar et al, 2022 (89)	Case-control	Patients with T2DM (N=112) *vs* Healthy control (N=112)	PRL in T2DM patients was significantly lower compared to healthy control. PRL levels were inversely correlated with total cholesterol, LDL-cholesterol, and TG, but not with HDL cholesterol.

MIC, microprolactinoma; MAC, macroprolactinoma; CAB, cabergoline; BRC, bromocriptine; DA, dopamine agonist; CRP, C reactive protein; PRL, prolactin; OR, odds ratio; CKI, chronic kidney insufficiency; T2DM, type 2 diabetes mellitus; FMD, flow mediated dilation; TG, triglycerides; AT-III, Antithrombin- III; PAI-1, plasminogen activator inhibitor 1; MRI, magnetic resonance imaging.

**Table 4:** Populational studies.

Authors, year	Study design	Population studied	Results
Haring et al, 2014 (166)	Population study	3929 individuals aged 20–81 yrs from the population-based Study of Health in Pomerania	Independent positive association of PRL levels with all-cause and cardiovascular mortality
Therkelsen et al, 2016 (94)	Population study (prospective)	3232 patients with normoprolactinemia were drawn from Framingham Heart Study, who attended 2 examinations 6 years apart	PRL was not associated with a CVD risk factor. In women, for each 5-mg/dL increment in PRL, increase OR for low HDL-cholesterol. In men, a 5-mg/dL increment in PRL was associated with increased OR of hypertension and diabetes
Krogh et al, 2017 (139)	Prospective Cohort	3633 individuals (mean age 39.7 years, 78% women)	373 patients developed hyperprolactinemia: there was an association between hyperprolactinemia and an increased risk of death from all causes and cardiovascular causes only in men

MIC, microprolactinoma; MAC, macroprolactinoma; CAB, cabergoline; BRC, bromocriptine; DA, dopamine agonist; CRP, C reactive protein; PRL, prolactin; OR, odds ratio; CKI, chronic kidney insufficiency; T2DM, type 2 diabetes mellitus; FMD, flow mediated dilation; TG, triglycerides; AT-III, Antithrombin- III; PAI-1, plasminogen activator inhibitor 1; MRI, magnetic resonance imaging.

A meta-analysis and a systematic review evaluating the effect of reducing PRL with DA on established cardiovascular risk factors in 387 patients with prolactinomas suggested, beyond the reduction in weight and BMI, a small decrease in waist circumference, a small-to-moderate decrease in triglycerides, fasting glucose levels, HOMA-IR, HbA1c and hs-CRP, and a moderate decrease in LDL, total cholesterol and insulin, however, data were considered of low-quality evidence (158). Noteworthy, clinical studies have shown that circadian-timed quick-release bromocriptine treatment reduces significantly the risk of major adverse cardiovascular events in type 2 diabetes mellitus, non-hyperprolactinemic patients (109, 169).Among other effects (e.g., improving insulin sensitivity), a potential sympatholytic mechanism has been proposed (170).

Therefore, well-designed studies will be necessary to strengthen or not those findings as well as a better understanding of the mechanism of association between PRL, DA, and cardiovascular risk.

## Conclusions

Hyperprolactinemia is classically associated with hypogonadism and reproductive symptoms. Beyond these effects, hyperprolactinemia presents recognized metabolic influences. However, the clinical impact of PRL role on metabolism and cardiovascular risk is not stablished.

Accordingly, no consistent data supports that DA treatment or normalization of PRL levels can prevent cardiovascular events (9). Studies suggested that DA treatment could improve CVD risk, either directly by normalizing serum PRL levels and reducing dopamine tone, or indirectly by restoring eugonadism. More studies evaluating CVD risk in hyperprolactinemic patients are important to define a potential indication of treatment beyond hypogonadism. Interestingly enough, low levels of PRL in population studies were associated with diabetes mellitus type 2 risk and metabolic syndrome (102). However, there are no data pointing to a negative influence of low serum PRL levels during DA treatment in glucose metabolism.

DA improves metabolic parameters such as glucose profile, and insulin resistance in patients with diabetes mellitus (171, 172) and patients with prolactinoma (38, 45). Cabergoline was able to improve metabolic parameters even without the correction of PRL excess (45, 48) or concomitant hypogonadism (47, 156). Similarly, both BRC and CAB have been demonstrated to significantly improve lipid profile independently of their impact on concomitant obesity (45, 47, 48, 156) and hypogonadism (47), leading to the hypothesis of a direct beneficial effect of DA on lipid profile (45, 47).

**Figure 5:**
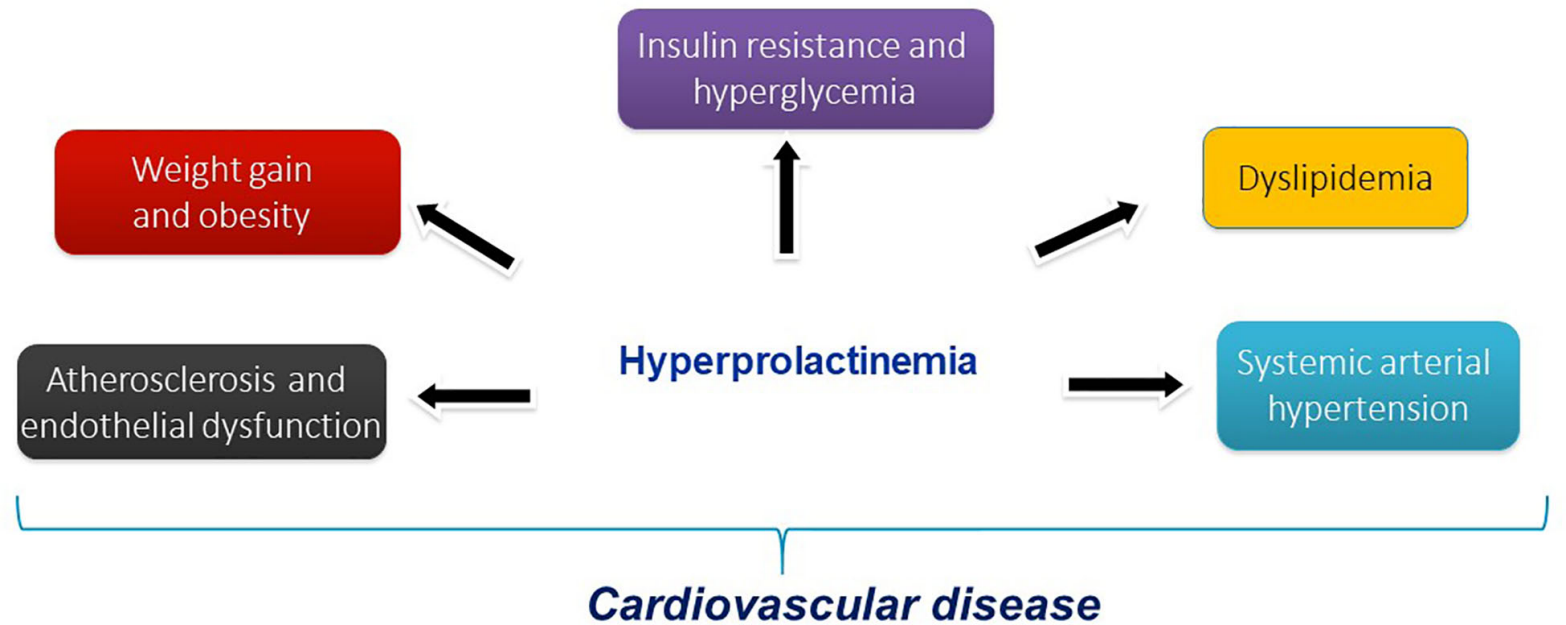
Hyperprolactinemia and cardiovascular risk factors.

In conclusion, hyperprolactinemia seems to be associated with insulin resistance and hyperglycemia, dyslipidemia, weight gain and obesity, systemic arterial hypertension, atherosclerosis and endothelial dysfunction increasing CVD (**Figure 5**). Additionally, hyperprolactinemia can be associated with metabolic abnormalities such as obesity, insulin resistance, and alterations in glucose metabolism that could accelerate atherosclerosis (173).

## Author contributions

All authors listed have made a substantial, direct, and intellectual contribution to the work, and approved it for publication.

## References

[1] De Zegher F Van Den Berghe G Devlieger H Eggermont E Veldhuis JD. Dopamine inhibits growth hormone and prolactin secretion in the human newborn. Pediatr Res (1993) 34(5):642–5. doi: 10.1203/00006450-199311000-00016 8284103

[2] Bole-Feysot C Goffin V Edery M Binart N Kelly PA. Prolactin (PRL) and its receptor: actions, signal transduction pathways and phenotypes observed in PRL receptor knockout mice. Endocr Rev (1998) 19(3):225–68. doi: 10.1210/edrv.19.3.0334 9626554

[3] Torner L. Actions of prolactin in the brain: From physiological adaptations to stress and neurogenesis to psychopathology. Front Endocrinol (Lausanne) (2016) 7:25. doi: 10.3389/fendo.2016.00025 27065946 PMC4811943

[4] Glezer A Soares CR Vieira JG Giannella-Neto D Ribela MT Goffin V. Human macroprolactin displays low biological activity *via* its homologous receptor in a new sensitive bioassay. J Clin Endocrinol Metab (2006) 91(3):1048–55. doi: 10.1210/jc.2005-1831 16384849

[5] Triebel J Robles-Osorio ML Garcia-Franco R Martínez de la Escalera G Clapp C Bertsch T. From bench to bedside: Translating the Prolactin/Vasoinhibin axis. Front Endocrinol (Lausanne) (2017) 8:342. doi: 10.3389/fendo.2017.00342 29321761 PMC5732132

[6] Melmed S Casanueva FF Hoffman AR Kleinberg DL Montori VM Schlechte JA. Diagnosis and treatment of hyperprolactinemia: an endocrine society clinical practice guideline. J Clin Endocrinol Metab (2011) 96(2):273–88. doi: 10.1210/jc.2010-1692 21296991

[7] Glezer A Bronstein MD. Prolactinomas. Endocrinol Metab Clin North Am (2015) 44(1):71–8. doi: 10.1016/j.ecl.2014.11.003 25732643

[8] Bonert V. Do nothing but observe microprolactinomas: when and how to replace sex hormones? Pituitary (2020) 23(3):307–13. doi: 10.1007/s11102-020-01039-x 32274622

[9] Greenman Y. Prolactinomas and menopause: any changes in management? Pituitary (2020) 23(1):58–64. doi: 10.1007/s11102-019-00998-0 31686376

[10] Roth GA Mensah GA Johnson CO Addolorato G Ammirati E Baddour LM. Global burden of cardiovascular diseases and risk factors, 1990-2019: Update from the GBD 2019 study. J Am Coll Cardiol (2020) 76(25):2982–3021. doi: 10.1016/j.jacc.2020.11.010 33309175 PMC7755038

[11] Mensah GA Roth GA Fuster V. The global burden of cardiovascular diseases and risk factors: 2020 and beyond. J Am Coll Cardiol (2019) 74(20):2529–32. doi: 10.1016/j.jacc.2019.10.009 31727292

[12] Bergheanu SC Bodde MC Jukema JW. Pathophysiology and treatment of atherosclerosis : Current view and future perspective on lipoprotein modification treatment. Neth Heart J (2017) 25(4):231–42. doi: 10.1007/s12471-017-0959-2 PMC5355390 28194698

[13] Ormazabal V Nair S Elfeky O Aguayo C Salomon C Zuñiga FA. Association between insulin resistance and the development of cardiovascular disease. Cardiovasc Diabetol (2018) 17(1):122. doi: 10.1186/s12933-018-0762-4 30170598 PMC6119242

[14] Bhupathiraju SN Hu FB. Epidemiology of obesity and diabetes and their cardiovascular complications. Circ Res (2016) 118(11):1723–35. doi: 10.1161/CIRCRESAHA.115.306825 PMC4887150 27230638

[15] Bays HE. Evaluation and practical management of increased visceral fat: Should cardiologists lose sleep over it? J Am Coll Cardiol (2022) 79(13):1266–9. doi: 10.1016/j.jacc.2022.01.039 35361349

[16] Daimon M Kamba A Murakami H Mizushiri S Osonoi S Yamaichi M. Association between serum prolactin levels and insulin resistance in non-diabetic men. PloS One (2017) 12(4):e0175204. doi: 10.1371/journal.pone.0175204 28384295 PMC5383244

[17] Pala NA Laway BA Misgar RA Dar RA. Metabolic abnormalities in patients with prolactinoma: response to treatment with cabergoline. Diabetol Metab Syndr (2015) 7:99. doi: 10.1186/s13098-015-0094-4 26583049 PMC4650139

[18] Greenman Y Tordjman K Stern N. Increased body weight associated with prolactin secreting pituitary adenomas: weight loss with normalization of prolactin levels. Clin Endocrinol (Oxf) (1998) 48(5):547–53. doi: 10.1046/j.1365-2265.1998.00403.x 9666865

[19] Naliato EC Violante AH Caldas D Lamounier Filho A Loureiro CR Fontes R. Body fat in nonobese women with prolactinoma treated with dopamine agonists. Clin Endocrinol (Oxf) (2007) 67(6):845–52. doi: 10.1111/j.1365-2265.2007.02973.x 17645576

[20] Breves JP Popp EE Rothenberg EF Rosenstein CW Maffett KM Guertin RR. Osmoregulatory actions of prolactin in the gastrointestinal tract of fishes. Gen Comp Endocrinol (2020) 298:113589. doi: 10.1016/j.ygcen.2020.113589 32827513

[21] Ziabreva EV Bol'shakova TD Gitel' EP Lukovnikova LP Podzolkov VI. [Role of prolactin in disorders of water and salt metabolism in patients with hypertension]. Kardiologiia (1986) 26(5):64–7. 3735922

[22] Horrobin DF Lloyd IJ Lipton A Burstyn PG Durkin N Muiruri KL. Actions of prolactin on human renal function. Lancet (1971) 2(7720):352–4. doi: 10.1016/s0140-6736(71)90065-1 4105050

[23] Buckman MT Peake GT Robertson G. Hyperprolactinemia influences renal function in man. Metabolism (1976) 25(5):509–16. doi: 10.1016/0026-0495(76)90004-4 1263843

[24] Tanaka S Shimamoto K Takada T Nakahashi Y Ando T Nishitani T. Plasma prolactin levels in patients with essential hypertension, malignant hypertension and secondary hypertension. Jpn J Med (1985) 24(1):19–23. doi: 10.2169/internalmedicine1962.24.19 3889434

[25] Oney T Bellmann O Kaulhausen H. Relationship between serum prolactin concentration, vascular angiotensin sensitivity and arterial blood pressure during third trimester pregnancy. Arch Gynecol Obstet (1988) 243(2):83–90. doi: 10.1007/BF00932973 3401043

[26] Zhang L Curhan GC Forman JP. Plasma prolactin level and risk of incident hypertension in postmenopausal women. J Hypertens (2010) 28(7):1400–5. doi: 10.1097/HJH.0b013e328339f254 PMC3139424 20453663

[27] Leaños-Miranda A Márquez-Acosta J Cárdenas-Mondragón GM Chinolla-Arellano ZL Rivera-Leaños R. Urinary prolactin as a reliable marker for preeclampsia, its severity, and the occurrence of adverse pregnancy outcomes. J Clin Endocrinol Metab (2008) 93(7):2492–9. doi: 10.1210/jc.2008-0305 18460570

[28] Leaños-Miranda A Campos-Galicia I Ramírez-Valenzuela KL Chinolla-Arellano ZL Isordia-Salas I. Circulating angiogenic factors and urinary prolactin as predictors of adverse outcomes in women with preeclampsia. Hypertension (2013) 61(5):1118–25. doi: 10.1161/HYPERTENSIONAHA.111.00754 23460287

[29] Alawad ZM Al-Omary HL. Maternal and cord blood prolactin level and pregnancy complications. Pak J Med Sci (2019) 35(4):1122–7. doi: 10.12669/pjms.35.4.558 PMC6659077 31372154

[30] Chang AS Grant R Tomita H Kim HS Smithies O Kakoki M. Prolactin alters blood pressure by modulating the activity of endothelial nitric oxide synthase. Proc Natl Acad Sci U.S.A. (2016) 113(44):12538–43. doi: 10.1073/pnas.1615051113 PMC5098649 27791173

[31] Zeng C Armando I Luo Y Eisner GM Felder RA Jose PA. Dysregulation of dopamine-dependent mechanisms as a determinant of hypertension: studies in dopamine receptor knockout mice. Am J Physiol Heart Circ Physiol (2008) 294(2):H551–69. doi: 10.1152/ajpheart.01036.2007 PMC4029502 18083900

[32] Georgiopoulos GA Stamatelopoulos KS Lambrinoudaki I Lykka M Kyrkou K Rizos D. Prolactin and preclinical atherosclerosis in menopausal women with cardiovascular risk factors. Hypertension (2009) 54(1):98–105. doi: 10.1161/HYPERTENSIONAHA.109.132100 19451414

[33] Koca AO Dagdeviren M Akkan T Keskin M Pamuk N Altay M. Is idiopathic mild hyperprolactinemia a cardiovascular risk factor? Niger J Clin Pract (2021) 24(2):213–9. doi: 10.4103/njcp.njcp_178_20 33605911

[34] Pelkonen R Nikkilä EA Grahne B. Serum lipids, postheparin plasma lipase activities and glucose tolerance in patients with prolactinoma. Clin Endocrinol (Oxf) (1982) 16(4):383–90. doi: 10.1111/j.1365-2265.1982.tb00731.x 7047001

[35] Erem C Kocak M Nuhoglu I Yılmaz M Ucuncu O. Blood coagulation, fibrinolysis and lipid profile in patients with prolactinoma. Clin Endocrinol (Oxf) (2010) 73(4):502–7. doi: 10.1111/j.1365-2265.2009.03752.x 20039901

[36] Oppenheim DS Greenspan SL Zervas NT Schoenfeld DA Klibanski A. Elevated serum lipids in hypogonadal men with and without hyperprolactinemia. Ann Intern Med (1989) 111(4):288–92. doi: 10.7326/0003-4819-111-4-288 2757313

[37] Perić B Kruljac I Šundalić S Pećina HI Jović A Štefanović M. Obesity and hypercholesterolemia in patients with prolactinomas: Could DHEA-s and growth hormone be the missing link? Endocr Res (2016) 41(3):200–6. doi: 10.3109/07435800.2015.1135444 26864960

[38] Berinder K Nystrom T Hoybye C Hall K Hulting AL. Insulin sensitivity and lipid profile in prolactinoma patients before and after normalization of prolactin by dopamine agonist therapy. Pituitary (2011) 14(3):199–207. doi: 10.1007/s11102-010-0277-9 21128120

[39] Krysiak R Okopien B. Different effects of cabergoline and bromocriptine on metabolic and cardiovascular risk factors in patients with elevated prolactin levels. Basic Clin Pharmacol Toxicol (2015) 116(3):251–6. doi: 10.1111/bcpt.12307 25123447

[40] Fahy U Hopton MI Hartog M Bolton CH Hull MG. The lipoprotein profile of women with hyperprolactinaemic amenorrhoea. Hum Reprod (1999) 14(2):285–7. doi: 10.1093/humrep/14.2.285 10099964

[41] Schwetz V Librizzi R Trummer C Theiler G Stiegler C Pieber TR. Treatment of hyperprolactinaemia reduces total cholesterol and LDL in patients with prolactinomas. Metab Brain Dis (2017) 32(1):155–61. doi: 10.1007/s11011-016-9882-2 PMC5566581 27525431

[42] Medic-Stojanoska M Icin T Pletikosic I Bajkin I Novakovic-Paro J Stokic E. Risk factors for accelerated atherosclerosis in young women with hyperprolactinemia. Med Hypotheses (2015) 84(4):321–6. doi: 10.1016/j.mehy.2015.01.024 25649851

[43] Inancli SS Usluogullari A Ustu Y Caner S Tam AA Ersoy R. Effect of cabergoline on insulin sensitivity, inflammation, and carotid intima media thickness in patients with prolactinoma. Endocrine (2013) 44(1):193–9. doi: 10.1007/s12020-012-9857-y 23233277

[44] Khalil G Khan FA Jamal QM Saleem A Masroor H Abbas K. Change in insulin sensitivity and lipid profile after dopamine agonist therapy in patients with prolactinoma. Cureus (2021) 13(9):e17824. doi: 10.7759/cureus.17824 34660034 PMC8505009

[45] Auriemma RS Granieri L Galdiero M Simeoli C Perone Y Vitale P. Effect of cabergoline on metabolism in prolactinomas. Neuroendocrinology (2013) 98(4):299–310. doi: 10.1159/000357810 24355865

[46] dos Santos Silva CM Barbosa FR Lima GA Warszawski L Fontes R Domingues RC. BMI and metabolic profile in patients with prolactinoma before and after treatment with dopamine agonists. Obes (Silver Spring) (2011) 19(4):800–5. doi: 10.1038/oby.2010.150 20559294

[47] Auriemma RS Galdiero M Vitale P. Effect of chronic cabergoline treatment and testosterone replacement on metabolism in male patients with prolactinomas. Neuroendocrinology (2015) 101(1):66–81. doi: 10.1159/000371851 25592453

[48] Ciresi A Amato MC Guarnotta V Lo Castro F Giordano C. Higher doses of cabergoline further improve metabolic parameters in patients with prolactinoma regardless of the degree of reduction in prolactin levels. Clin Endocrinol (Oxf) (2013) 79(6):845–52. doi: 10.1111/cen.12204 23506485

[49] Posawetz AS Trummer C Pandis M Aberer F Pieber TR Obermayer-Pietsch B. Adverse body composition and lipid parameters in patients with prolactinoma: A case-control study. BMC Endocr Disord (2021) 21(1):81. doi: 10.1186/s12902-021-00733-6 33902531 PMC8074459

[50] Jiang XB Li CL He DS Mao ZG Liu DH Fan X. Increased carotid intima media thickness is associated with prolactin levels in subjects with untreated prolactinoma: A pilot study. Pituitary (2014) 17(3):232–9. doi: 10.1007/s11102-013-0495-z 23756783

[51] Heshmati HM Turpin G de Gennes JL. Chronic hyperprolactinemia and plasma lipids in women. Klin Wochenschr (1987) 65(11):516–9. doi: 10.1007/BF01721038 3613464

[52] Ling C Svensson L Odén B Weijdegård B Edén B Edén S. Identification of functional prolactin (PRL) receptor gene expression: PRL inhibits lipoprotein lipase activity in human white adipose tissue. J Clin Endocrinol Metab (2003) 88(4):1804–8. doi: 10.1210/jc.2002-021137 12679477

[53] Kamath V Jones CN Yip JC Varasteh BB Cincotta AH. Effects of a quick-release form of bromocriptine (Ergoset) on fasting and postprandial plasma glucose, insulin, lipid, and lipoprotein concentrations in obese nondiabetic hyperinsulinemic women. Diabetes Care (1997) 20(11):1697–701. doi: 10.2337/diacare.20.11.1697 9353611

[54] Pirchio R Auriemma RS Solari D Arnesi M Pivonello C Negri M. Effects of pituitary surgery and high-dose cabergoline therapy on metabolic profile in patients with prolactinoma resistant to conventional cabergoline treatment. Front Endocrinol (Lausanne) (2021) 12:769744. doi: 10.3389/fendo.2021.769744 34917030 PMC8670228

[55] Krysiak R Szkróbka W Okopień B. Different effects of atorvastatin on cardiometabolic risk factors in young women with and without hyperprolactinemia. J Clin Pharmacol (2019) 59(1):83–9. doi: 10.1002/jcph.1301 30129670

[56] Krysiak R Marek B Okopień B. Cardiometabolic risk factors in young women with macroprolactinaemia. Endokrynol Pol (2019) 70(4):336–41. doi: 10.5603/EP.a2019.0013 30845340

[57] Krysiak R Kowalcze K Okopień B. Impact of macroprolactinemia on cardiometabolic effects of atorvastatin in women with hypercholesterolemia. Am J Cardiol (2019) 124(8):1207–12. doi: 10.1016/j.amjcard.2019.07.017 31409451

[58] Krysiak R Szkróbka W Okopień B. Cardiometabolic risk factors in men with elevated macroprolactin content: A pilot study. Exp Clin Endocrinol Diabetes (2021) 129(1):7–13. doi: 10.1055/a-0902-4439 31185509

[59] Arslan MS Topaloglu O Sahin M Tutal E Gungunes A Cakir E. Preclinical atherosclerosis in patients with prolactinoma. Endocr Pract (2014) 20(5):447–51. doi: 10.4158/EP13173.OR 24325995

[60] Reuwer AQ van Eijk M Houttuijn-Bloemendaal FM van der Loos CM Claessen N Teeling P. The prolactin receptor is expressed in macrophages within human carotid atherosclerotic plaques: A role for prolactin in atherogenesis? J Endocrinol (2011) 208(2):107–17. doi: 10.1677/JOE-10-0076 21068074

[61] Goldhar AS Vonderhaar BK Trott JF Hovey RC. Prolactin-induced expression of vascular endothelial growth factor *via* egr-1. Mol Cell Endocrinol (2005) 232(1-2):9–19. doi: 10.1016/j.mce.2005.01.005 15737464

[62] Molinari C Grossini E Mary DA Uberti F Ghigo E Ribichini F. Prolactin induces regional vasoconstriction through the beta2-adrenergic and nitric oxide mechanisms. Endocrinology (2007) 148(8):4080–90. doi: 10.1210/en.2006-1577 17463060

[63] Sauro MD Bing B Zorn NE. Prolactin induces growth-related gene expression in rat aortic smooth muscle in vivo. Eur J Pharmacol (1992) 225(4):351–4. doi: 10.1016/0922-4106(92)90110-h 1499663

[64] Yavuz D Deyneli O Akpinar I Yildiz E Gözü H Sezgin O. Endothelial function, insulin sensitivity and inflammatory markers in hyperprolactinemic pre-menopausal women. Eur J Endocrinol (2003) 149(3):187–93. doi: 10.1530/eje.0.1490187 12943520

[65] Doğan BA Arduç A Tuna MM Nasıroğlu NI Işık S Berker D. Evaluation of atherosclerosis after cessation of cabergoline therapy in patients with prolactinoma. Anatol J Cardiol (2016) 16(6):440–7. doi: 10.5152/AnatolJCardiol.2015.6416 PMC5331378 26680550

[66] Yazici D Sunbul M Yasar M Deyneli O Yavuz D. Is there an increased cardiovascular risk in patients with prolactinoma? a challenging question. J Clin Ultrasound (2021) 49(8):870–7. doi: 10.1002/jcu.23030 34131923

[67] Serri O Li L Mamputu JC Beauchamp MC Maingrette F Renier G. The influences of hyperprolactinemia and obesity on cardiovascular risk markers: effects of cabergoline therapy. Clin Endocrinol (Oxf) (2006) 64(4):366–70. doi: 10.1111/j.1365-2265.2006.02469.x 16584506

[68] Krysiak R Gilowski W Szkrobka W Okopien B. The effect of atorvastatin on cardiometabolic risk factors in bromocriptine-treated premenopausal women with isolated hypercholesterolemia. Cardiovasc Ther (2015) 33(5):282–7. doi: 10.1111/1755-5922.12143 26146893

[69] Iacobellis G Barbaro G. The double role of epicardial adipose tissue as pro- and anti-inflammatory organ. Horm Metab Res (2008) 40(7):442–5. doi: 10.1055/s-2008-1062724 18401833

[70] Soto-Pedre E Newey PJ Bevan JS Leese GP. Morbidity and mortality in patients with hyperprolactinaemia: the PROLEARS study. Endocr Connect (2017) 6(8):580–8. doi: 10.1530/EC-17-0171 PMC5633062 28954743

[71] Toulis KA Robbins T Reddy N Balachandran K Gokhale K Wijesinghe H. Males with prolactinoma are at increased risk of incident cardiovascular disease. Clin Endocrinol (Oxf) (2018) 88(1):71–6. doi: 10.1111/cen.13498 29044586

[72] Freemark M Avril I Fleenor D Driscoll P Petro A Opara E. Targeted deletion of the PRL receptor: effects on islet development, insulin production, and glucose tolerance. Endocrinology (2002) 143(4):1378–85. doi: 10.1210/endo.143.4.8722 11897695

[73] Ben-Jonathan N Hugo ER Brandebourg TD LaPensee CR. Focus on prolactin as a metabolic hormone. Trends Endocrinol Metab (2006) 17(3):110–6. doi: 10.1016/j.tem.2006.02.005 16517173

[74] Sorenson RL Stout LE. Prolactin receptors and JAK2 in islets of langerhans: an immunohistochemical analysis. Endocrinology (1995) 136(9):4092–8. doi: 10.1210/endo.136.9.7649117 7649117

[75] Li M Song Y Rawal S Hinkle SN Zhu Y Tekola-Ayele F. Plasma prolactin and progesterone levels and the risk of gestational diabetes: A prospective and longitudinal study in a multiracial cohort. Front Endocrinol (Lausanne) (2020) 11:83. doi: 10.3389/fendo.2020.00083 32180760 PMC7058109

[76] Ekinci EI Torkamani N Ramchand SK Churilov L Sikaris KA Lu ZX. Higher maternal serum prolactin levels are associated with reduced glucose tolerance during pregnancy. J Diabetes Investig (2017) 8(5):697–700. doi: 10.1111/jdi.12634 PMC5583956 28129477

[77] Le TN Elsea SH Romero R Chaiworapongsa T Francis GL. Prolactin receptor gene polymorphisms are associated with gestational diabetes. Genet Test Mol Biomarkers (2013) 17(7):567–71. doi: 10.1089/gtmb.2013.0009 PMC3700434 23651351

[78] Overgaard M Glintborg D Christesen HT Jensen TK Andersen MS. Maternal prolactin is associated with glucose status and PCOS in pregnancy: Odense child cohort. Eur J Endocrinol (2020) 183(3):307–16. doi: 10.1530/EJE-20-0144 32570208

[79] Ozisik H Suner A Cetinkalp S. Prolactin effect on blood glucose and insülin in breastfeeding women. Diabetes Metab Syndr (2019) 13(3):1765–7. doi: 10.1016/j.dsx.2019.03.045 31235091

[80] Gunderson EP Lewis CE Lin Y Sorel M Gross M Sidney S. Lactation duration and progression to diabetes in women across the childbearing years: The 30-year CARDIA study. JAMA Intern Med (2018) 178(3):328–37. doi: 10.1001/jamainternmed.2017.7978 PMC5885916 29340577

[81] Zhang Z Piro AL Allalou A Alexeeff SE Dai FF Gunderson EP. Prolactin and maternal metabolism in women with a recent GDM pregnancy and links to future T2D: The SWIFT study. J Clin Endocrinol Metab (2022) 107(9):2652–65. doi: 10.1210/clinem/dgac346 PMC9387721 35666146

[82] Labriola L Montor WR Krogh K Lojudice FH Genzini T Goldberg AC. Beneficial effects of prolactin and laminin on human pancreatic islet-cell cultures. Mol Cell Endocrinol (2007) 263(1-2):120–33. doi: 10.1016/j.mce.2006.09.011 17081683

[83] Fleenor DE Freemark M. Prolactin induction of insulin gene transcription: roles of glucose and signal transducer and activator of transcription 5. Endocrinology (2001) 142(7):2805–10. doi: 10.1210/endo.142.7.8267 11415999

[84] Cejkova P Fojtikova M Cerna M. Immunomodulatory role of prolactin in diabetes development. Autoimmun Rev (2009) 9(1):23–7. doi: 10.1016/j.autrev.2009.02.031 19248843

[85] Wang T Lu J Xu Y Li M Sun J Zhang J. Circulating prolactin associates with diabetes and impaired glucose regulation: A population-based study. Diabetes Care (2013) 36(7):1974–80. doi: 10.2337/dc12-1893 PMC3687322 23340889

[86] Chahar C Chahar K Ankit BS Gadhwal A Agrawal RP. Association of serum prolactin level with impaired glucose regulation and diabetes. J Assoc Physicians India (2017) 65(3):34–9. 28462541

[87] Ruiz-Herrera X de Los Ríos EA Díaz JM Lerma-Alvarado RM Martínez de la Escalera L. Prolactin promotes adipose tissue fitness and insulin sensitivity in obese males. Endocrinology (2017) 158(1):56–68. doi: 10.1210/en.2016-1444 27805870

[88] Wagner R Heni M Linder K Ketterer C Peter A Böhm A. Age-dependent association of serum prolactin with glycaemia and insulin sensitivity in humans. Acta Diabetol (2014) 51(1):71–8. doi: 10.1007/s00592-013-0493-7 23836327

[89] Jayashankar CA Manohar A Joshi A Dwarakanathan V Pinnelli VBK Sarathi V. Association of serum prolactin with type 2 diabetes mellitus: A comparative cross-sectional study from south India. Cureus (2022) 14(4):e23721. doi: 10.7759/cureus.23721 35509763 PMC9060740

[90] Balbach L Wallaschofski H Völzke H Nauck M Dörr M Haring R. Serum prolactin concentrations as risk factor of metabolic syndrome or type 2 diabetes? BMC Endocr Disord (2013) 13:12. doi: 10.1186/1472-6823-13-12 23517652 PMC3614874

[91] Li J Rice MS Huang T Hankinson SE Clevenger CV Hu FB. Circulating prolactin concentrations and risk of type 2 diabetes in US women. Diabetologia (2018) 61(12):2549–60. doi: 10.1007/s00125-018-4733-9 PMC6309828 30306190

[92] Wang T Xu Y Xu M Ning G Lu J Dai M. Circulating prolactin and risk of type 2 diabetes: A prospective study. Am J Epidemiol (2016) 184(4):295–301. doi: 10.1093/aje/kwv326 27466075

[93] Retnakaran R Ye C Kramer CK Connelly PW Hanley AJ Sermer M. Maternal serum prolactin and prediction of postpartum β-cell function and risk of Prediabetes/Diabetes. Diabetes Care (2016) 39(7):1250–8. doi: 10.2337/dc16-0043 27208323

[94] Therkelsen KE Abraham TM Pedley A Massaro JM Sutherland P Hoffmann U. Association between prolactin and incidence of cardiovascular risk factors in the framingham heart study. J Am Heart Assoc (2016) 5(2). doi: 10.1161/JAHA.115.002640 PMC4802489 26908403

[95] Faria de Castro L Alves Dos Santos Á Augusto Casulari L Ansaneli Naves L Amorim Amato A. Association between variations of physiological prolactin serum levels and the risk of type 2 diabetes: A systematic review and meta-analysis. Diabetes Res Clin Pract (2020) 166:108247. doi: 10.1016/j.diabres.2020.108247 32505717

[96] Landgraf R Landraf-Leurs MM Weissmann A Horl R von Werder K Scriba PC. Prolactin: a diabetogenic hormone. Diabetologia (1977) 13(2):99–104. doi: 10.1007/BF00745135 852641

[97] Tuzcu A Yalaki S Arikan S Gokalp D Bahcec M Tuzcu S. Evaluation of insulin sensitivity in hyperprolactinemic subjects by euglycemic hyperinsulinemic clamp technique. Pituitary (2009) 12(4):330–4. doi: 10.1007/s11102-009-0183-1 19408128

[98] Ratner LD Stevens G Bonaventura MM Lux-Lantos VA Poutanen M Calandra RS. Hyperprolactinemia induced by hCG leads to metabolic disturbances in female mice. J Endocrinol (2016) 230(1):157–69. doi: 10.1530/JOE-15-0528 27154336

[99] Park S Kim DS Daily JW Kim SH. Serum prolactin concentrations determine whether they improve or impair β-cell function and insulin sensitivity in diabetic rats. Diabetes Metab Res Rev (2011) 27(6):564–74. doi: 10.1002/dmrr.1215 21557442

[100] Park S Kang S Lee HW Ko BS. Central prolactin modulates insulin sensitivity and insulin secretion in diabetic rats. Neuroendocrinology (2012) 95(4):332–43. doi: 10.1159/000336501 22441304

[101] Doknic M Pekic S Zarkovic M Medic-Stojanoska M Dieguez C Casanueva F. Dopaminergic tone and obesity: an insight from prolactinomas treated with bromocriptine. Eur J Endocrinol (2002) 147(1):77–84. doi: 10.1530/eje.0.1470077 12088923

[102] Macotela Y Triebel J Clapp C. Time for a new perspective on prolactin in metabolism. Trends Endocrinol Metab (2020) 31(4):276–86. doi: 10.1016/j.tem.2020.01.004 32044206

[103] Corona G Rastrelli G Boddi V Monami M Melani C Balzi D. Prolactin levels independently predict major cardiovascular events in patients with erectile dysfunction. Int J Androl (2011) 34(3):217–24. doi: 10.1111/j.1365-2605.2010.01076.x 20522124

[104] Cincotta AH Schiller BC Landry RJ Herbert SJ Miers WR Meier AH. Circadian neuroendocrine role in age-related changes in body fat stores and insulin sensitivity of the male sprague-dawley rat. Chronobiol Int (1993) 10(4):244–58. doi: 10.1080/07420529309059707 8403068

[105] Luo S Zhang Y Ezrokhi M Li Y Tsai TH Cincotta AH. Circadian peak dopaminergic activity response at the biological clock pacemaker (suprachiasmatic nucleus) area mediates the metabolic responsiveness to a high-fat diet. J Neuroendocrinol (2018) 30(1). doi: 10.1111/jne.12563 PMC5817247 29224246

[106] Andersen IB Andreassen M Krogh J. The effect of dopamine agonists on metabolic variables in adults with type 2 diabetes: A systematic review with meta analysis and trial sequential analysis of randomized clinical trials. Diabetes Obes Metab (2021) 23(1):58–67. doi: 10.1111/dom.14183 32869474

[107] Bell DS. Why does quick-release bromocriptine decrease cardiac events? Diabetes Obes Metab (2011) 13(10):880–4. doi: 10.1111/j.1463-1326.2011.01424.x 21569186

[108] Cincotta AH Cersosimo E Alatrach M Ezrokhi M Agyin C Adams J. Bromocriptine-QR therapy reduces sympathetic tone and ameliorates a pro-Oxidative/Pro-Inflammatory phenotype in peripheral blood mononuclear cells and plasma of type 2 diabetes subjects. Int J Mol Sci (2022) 23(16). doi: 10.3390/ijms23168851 PMC9407769 36012132

[109] Ter Horst KW Lammers NM Trinko R Opland DM Figee M Ackermans MT. Striatal dopamine regulates systemic glucose metabolism in humans and mice. Sci Transl Med (2018) 10(442). doi: 10.1126/scitranslmed.aar3752 29794060

[110] Caravaggio F Borlido C Hahn M Feng Z Fervaha G Gerretsen P. Reduced insulin sensitivity is related to less endogenous dopamine at D2/3 receptors in the ventral striatum of healthy nonobese humans. Int J Neuropsychopharmacol (2015) 18(7):pyv014. doi: 10.1093/ijnp/pyv014 25716779 PMC4540108

[111] Lopez-Vicchi F De Winne C Ornstein AM Sorianello E Toneatto J Becu-Villalobos D. Severe hyperprolactinemia promotes brown adipose tissue whitening and aggravates high fat diet induced metabolic imbalance. Front Endocrinol (Lausanne) (2022) 13:883092. doi: 10.3389/fendo.2022.883092 35757410 PMC9226672

[112] Lopez-Vicchi F De Winne C Brie B Sorianello E Ladyman SR Becu-Villalobos D. Metabolic functions of prolactin: Physiological and pathological aspects. J Neuroendocrinol (2020) 32(11):e12888. doi: 10.1111/jne.12888 33463813

[113] Lopez-Vicchi F Ladyman SR Ornstein AM Gustafson P Knowles P Luque GM. Chronic high prolactin levels impact on gene expression at discrete hypothalamic nuclei involved in food intake. FASEB J (2020) 34(3):3902–14. doi: 10.1096/fj.201902357R 31944423

[114] Shibli-Rahhal A Schlechte J. The effects of hyperprolactinemia on bone and fat. Pituitary (2009) 12(2):96–104. doi: 10.1007/s11102-008-0097-3 18338266

[115] Luque GM Lopez-Vicchi F Ornstein AM Brie B De Winne C Fiore E. Chronic hyperprolactinemia evoked by disruption of lactotrope dopamine D2 receptors impacts on liver and adipocyte genes related to glucose and insulin balance. Am J Physiol Endocrinol Metab (2016) 311(6):E974–88. doi: 10.1152/ajpendo.00200.2016 27802964

[116] Reinholz J Skopp O Breitenstein C Bohr I Winterhoff H Knecht S. Compensatory weight gain due to dopaminergic hypofunction: new evidence and own incidental observations. Nutr Metab (Lond) (2008) 5:35. doi: 10.1186/1743-7075-5-35 19046419 PMC2615020

[117] Nilsson L Binart N Bohlooly-Y M Brie B De Winne C Fiore E. Prolactin and growth hormone regulate adiponectin secretion and receptor expression in adipose tissue. Biochem Biophys Res Commun (2005) 331(4):1120–6. doi: 10.1016/j.bbrc.2005.04.026 15882993

[118] Asai-Sato M Okamoto M Endo M Yoshida H Murase M Ikeda M. Hypoadiponectinemia in lean lactating women: Prolactin inhibits adiponectin secretion from human adipocytes. Endocr J (2006) 53(4):555–62. doi: 10.1507/endocrj.k06-026 16849835

[119] Mendoza-Herrera K Florio AA Moore M Marrero A Tamez M Bhupathiraju SN. The leptin system and diet: A mini review of the current evidence. Front Endocrinol (Lausanne) (2021) 12:749050. doi: 10.3389/fendo.2021.749050 34899599 PMC8651558

[120] Grove JCR Gray LA La Santa Medina N Sivakumar N Ahn JS Corpuz TV. Dopamine subsystems that track internal states. Nature (2022) 608(7922):374–80. doi: 10.1038/s41586-022-04954-0 PMC9365689 35831501

[121] Opland DM Leinninger GM Myers MG. Modulation of the mesolimbic dopamine system by leptin. Brain Res (2010) 1350:65–70. doi: 10.1016/j.brainres.2010.04.028 20417193 PMC2921997

[122] Carvalho JC Lisboa PC de Oliveira E Peixoto-Silva N Pinheiro CR Fraga MC. Effects of early and late neonatal bromocriptine treatment on hypothalamic neuropeptides, dopaminergic reward system and behavior of adult rats. Neuroscience (2016) 325:175–87. doi: 10.1016/j.neuroscience.2016.03.046 27038750

[123] Sun R Sugiyama M Wang S Kuno M Sasaki T Hirose T. Inflammation in VTA caused by HFD induces activation of dopaminergic neurons accompanied by binge-like eating. Nutrients (2022) 14(18). doi: 10.3390/nu14183835 PMC9502544 36145208

[124] Perez-Bonilla P Santiago-Colon K Leinninger GM. Lateral hypothalamic area neuropeptides modulate ventral tegmental area dopamine neurons and feeding. Physiol Behav (2020) 223:112986. doi: 10.1016/j.physbeh.2020.112986 32492498 PMC7416562

[125] Leenaerts N Jongen D Ceccarini J Van Oudenhove L Vrieze E. The neurobiological reward system and binge eating: A critical systematic review of neuroimaging studies. Int J Eat Disord (2022). doi: 10.1002/eat.23776 35841198

[126] Missale C Nash SR Robinson SW Jaber M Caron MG. Dopamine receptors: from structure to function. Physiol Rev (1998) 78(1):189–225. doi: 10.1152/physrev.1998.78.1.189 9457173

[127] Leinninger GM Jo YH Leshan RL Louis GW Yang H Barrera JG. Leptin acts *via* leptin receptor-expressing lateral hypothalamic neurons to modulate the mesolimbic dopamine system and suppress feeding. Cell Metab (2009) 10(2):89–98. doi: 10.1016/j.cmet.2009.06.011 19656487 PMC2723060

[128] Kullmann S Blum D Jaghutriz BA Gassenmaier C Bender B Häring HU. Central insulin modulates dopamine signaling in the human striatum. J Clin Endocrinol Metab (2021) 106(10):2949–61. doi: 10.1210/clinem/dgab410 34131733

[129] Pan X Zhang M Tian A Chen L Sun Z Wang L. Exploring the genetic correlation between obesity-related traits and regional brain volumes: Evidence from UK biobank cohort. NeuroImage Clin (2022) 33:102870. doi: 10.1016/j.nicl.2021.102870 34872017 PMC8648807

[130] Wang GJ Volkow ND Logan J Pappas NR Wong CT Zhu W. Brain dopamine and obesity. Lancet (2001) 357(9253):354–7. doi: 10.1016/s0140-6736(00)03643-6 11210998

[131] Eisenstein SA Bischoff AN Gredysa DM Antenor-Dorsey JA Koller JM Al-Lozi A. Emotional eating phenotype is associated with central dopamine D2 receptor binding independent of body mass index. Sci Rep (2015) 5:11283. doi: 10.1038/srep11283 26066863 PMC4464302

[132] Janssen LK Horstmann A. Molecular imaging of central dopamine in obesity: A qualitative review across substrates and radiotracers. Brain Sci (2022) 12(4). doi: 10.3390/brainsci12040486 PMC9031606 35448017

[133] Liu S Borgland SL. Insulin actions in the mesolimbic dopamine system. Exp Neurol (2019) 320:113006. doi: 10.1016/j.expneurol.2019.113006 31279911

[134] Baptista T Lacruz A de Mendoza S Mendoza Guillén JM Silvera R. Body weight gain after administration of antipsychotic drugs: correlation with leptin, insulin and reproductive hormones. Pharmacopsychiatry (2000) 33(3):81–8. doi: 10.1055/s-2000-8451 10855458

[135] Colao A Sarno AD Cappabianca P Briganti F Pivonello R Somma CD. Gender differences in the prevalence, clinical features and response to cabergoline in hyperprolactinemia. Eur J Endocrinol (2003) 148(3):325–31. doi: 10.1530/eje.0.1480325 12611613

[136] Strader AD Buntin JD. Changes in agouti-related peptide during the ring dove breeding cycle in relation to prolactin and parental hyperphagia. J Neuroendocrinol (2003) 15(11):1046–53. doi: 10.1046/j.1365-2826.2003.01092.x 14622434

[137] Bina KG Cincotta AH. Dopaminergic agonists normalize elevated hypothalamic neuropeptide y and corticotropin-releasing hormone, body weight gain, and hyperglycemia in ob/ob mice. Neuroendocrinology (2000) 71(1):68–78. doi: 10.1159/000054522 10644901

[138] Auriemma RS De Alcubierre D Pirchio R Pivonello R Colao A. The effects of hyperprolactinemia and its control on metabolic diseases. Expert Rev Endocrinol Metab (2018) 13(2):99–106. doi: 10.1080/17446651.2018.1434412 30058862

[139] Krogh J Selmer C Torp-Pedersen C Gislason GH Kistorp C. Hyperprolactinemia and the association with all-cause mortality and cardiovascular mortality. Horm Metab Res (2017) 49(6):411–7. doi: 10.1055/s-0043-107243 28437810

[140] Rangel N Villegas VE Rondón-Lagos M. Obesity and androgen receptor signaling: Associations and potential crosstalk in breast cancer cells. Cancers (Basel) (2021) 13(9). doi: 10.3390/cancers13092218 PMC8125357 34066328

[141] Varlamov O White AE Carroll JM Bethea CL Reddy A Slayden O. Androgen effects on adipose tissue architecture and function in nonhuman primates. Endocrinology (2012) 153(7):3100–10. doi: 10.1210/en.2011-2111 PMC3380299 22547568

[142] Stewart WC Baugh JE Floyd ZE Stephens JM. STAT 5 activators can replace the requirement of FBS in the adipogenesis of 3T3-L1 cells. Biochem Biophys Res Commun (2004) 324(1):355–9. doi: 10.1016/j.bbrc.2004.09.053 15465026

[143] Auffret J Viengchareun S Carre N Denis RG Magnan C Marie PY. Beige differentiation of adipose depots in mice lacking prolactin receptor protects against high-fat-diet-induced obesity. FASEB J (2012) 26(9):3728–37. doi: 10.1096/fj.12-204958 22637534

[144] Gualillo O Lago F García M Menéndez C Señarís R Casanueva FF. Prolactin stimulates leptin secretion by rat white adipose tissue. Endocrinology (1999) 140(11):5149–53. doi: 10.1210/endo.140.11.7147 10537143

[145] Zinger M McFarland M Ben-Jonathan N. Prolactin expression and secretion by human breast glandular and adipose tissue explants. J Clin Endocrinol Metab (2003) 88(2):689–96. doi: 10.1210/jc.2002-021255 12574200

[146] Kok P Roelfsema F Frölich M Meinders AE Pijl H. Prolactin release is enhanced in proportion to excess visceral fat in obese women. J Clin Endocrinol Metab (2004) 89(9):4445–9. doi: 10.1210/jc.2003-032184 15356045

[147] Khurana S Kuns R Ben-Jonathan N. Heparin-binding property of human prolactin: a novel aspect of prolactin biology. Endocrinology (1999) 140(2):1026–9. doi: 10.1210/endo.140.2.6677 9927340

[148] Liu J Zhang L Fu J Wang Q Wang G. Circulating prolactin level is increased in metabolically healthy obesity. Endocr Connect (2021) 10(4):484–91. doi: 10.1530/EC-21-0040 PMC8111314 33794504

[149] Delgrange E Donckier J Maiter D. Hyperprolactinaemia as a reversible cause of weight gain in male patients? Clin Endocrinol (Oxf) (1999) 50(2):271. doi: 10.1046/j.1365-2265.1999.00700.x 10396373

[150] Grattan DR. 60 years of neuroendocrinology: The hypothalamo-prolactin axis. J Endocrinol (2015) 226(2):T101–22. doi: 10.1530/JOE-15-0213 PMC4515538 26101377

[151] Gerardo-Gettens T Moore BJ Stern JS Horwitz BA. Prolactin stimulates food intake in a dose-dependent manner. Am J Physiol (1989) 256(1 Pt 2):R276–80. doi: 10.1152/ajpregu.1989.256.1.R276 2912221

[152] Sauvé D Woodside B. The effect of central administration of prolactin on food intake in virgin female rats is dose-dependent, occurs in the absence of ovarian hormones and the latency to onset varies with feeding regimen. Brain Res (1996) 729(1):75–81. doi: 10.1016/S0006-8993(96)00227-2 8874878

[153] Sauvé D Woodside B. Neuroanatomical specificity of prolactin-induced hyperphagia in virgin female rats. Brain Res (2000) 868(2):306–14. doi: 10.1016/s0006-8993(00)02344-1 10854583

[154] Naef L Woodside B. Prolactin/Leptin interactions in the control of food intake in rats. Endocrinology (2007) 148(12):5977–83. doi: 10.1210/en.2007-0442 17872372

[155] Altuntaş S Evran M Sert M Tetiker T. Markers of metabolic syndrome in patients with pituitary adenoma: A case series of 303 patients. Horm Metab Res (2019) 51(11):709–13. doi: 10.1055/a-1020-3992 31683340

[156] Naliato EC Violante AH Gaccione M Caldas D Lamounier Filho A Loureiro CR. Body fat in men with prolactinoma. J Endocrinol Invest (2008) 31(11):985–90. doi: 10.1007/BF03345636 19169054

[157] Korner J Lo J Freda PU Wardlaw SL. Treatment with cabergoline is associated with weight loss in patients with hyperprolactinemia. Obes Res (2003) 11(2):311–2. doi: 10.1038/oby.2003.46 12582229

[158] Byberg S Futtrup J Andreassen M Krogh J. Metabolic effects of dopamine agonists in patients with prolactinomas: A systematic review and meta-analysis. Endocr Connect (2019) 8(10):1395–404. doi: 10.1530/EC-19-0286 PMC6826167 31518995

[159] Goland S Modi K Bitar F Janmohamed M Mirocha JM Czer LS. Clinical profile and predictors of complications in peripartum cardiomyopathy. J Card Fail (2009) 15(8):645–50. doi: 10.1016/j.cardfail.2009.03.008 19786252

[160] Arany Z Elkayam U. Peripartum cardiomyopathy. Circulation (2016) 133(14):1397–409. doi: 10.1161/CIRCULATIONAHA.115.020491 27045128

[161] Honigberg MC Givertz MM. Arrhythmias in peripartum cardiomyopathy. Card Electrophysiol Clin (2015) 7(2):309–17. doi: 10.1016/j.ccep.2015.03.010 26002395

[162] Sliwa K Blauwet L Tibazarwa K Libhaber E Smedema JP Becker A. Evaluation of bromocriptine in the treatment of acute severe peripartum cardiomyopathy: A proof-of-concept pilot study. Circulation (2010) 121(13):1465–73. doi: 10.1161/CIRCULATIONAHA.109.901496 20308616

[163] Hilfiker-Kleiner D Haghikia A Berliner D Vogel-Claussen J Schwab J Franke A. Bromocriptine for the treatment of peripartum cardiomyopathy: A multicentre randomized study. Eur Heart J (2017) 38(35):2671–9. doi: 10.1093/eurheartj/ehx355 PMC5837241 28934837

[164] Regitz-Zagrosek V Roos-Hesselink JW Bauersachs J Blomström-Lundqvist C Cífková R De Bonis M. 2018 ESC Guidelines for the management of cardiovascular diseases during pregnancy. Eur Heart J (2018) 39(34):3165–241. doi: 10.1093/eurheartj/ehy340 30165544

[165] Carrero JJ Kyriazis J Sonmez A Tzanakis I Qureshi AR Stenvinkel P. Prolactin levels, endothelial dysfunction, and the risk of cardiovascular events and mortality in patients with CKD. Clin J Am Soc Nephrol (2012) 7(2):207–15. doi: 10.2215/CJN.06840711 PMC3280028 22193237

[166] Haring R Friedrich N Völzke H Vasan RS Felix SB Dörr M. Positive association of serum prolactin concentrations with all-cause and cardiovascular mortality. Eur Heart J (2014) 35(18):1215–21. doi: 10.1093/eurheartj/ehs233 22843444

[167] Dourado M Cavalcanti F Vilar L Cantilino A. Relationship between prolactin, chronic kidney disease, and cardiovascular risk. Int J Endocrinol (2020) 2020:9524839. doi: 10.1155/2020/9524839 32655635 PMC7327580

[168] Reuwer AQ Twickler MT Hutten BA Molema FW Wareham NJ Dallinga-Thie GM. Prolactin levels and the risk of future coronary artery disease in apparently healthy men and women. Circ Cardiovasc Genet (2009) 2(4):389–95. doi: 10.1161/CIRCGENETICS.109.853572 20031611

[169] Gaziano JM Cincotta AH Vinik A Blonde L Bohannon N Scranton R. Effect of bromocriptine-QR (a quick-release formulation of bromocriptine mesylate) on major adverse cardiovascular events in type 2 diabetes subjects. J Am Heart Assoc (2012) 1(5):e002279. doi: 10.1161/JAHA.112.002279 23316290 PMC3541616

[170] Chamarthi B Vinik A Ezrokhi M Cincotta AH. Circadian-timed quick-release bromocriptine lowers elevated resting heart rate in patients with type 2 diabetes mellitus. Endocrinol Diabetes Metab (2020) 3(1):e00101. doi: 10.1002/edm2.101 31922028 PMC6947713

[171] Pijl H Ohashi S Matsuda M Miyazaki Y Mahankali A Kumar V. Bromocriptine: a novel approach to the treatment of type 2 diabetes. Diabetes Care (2000) 23(8):1154–61. doi: 10.2337/diacare.23.8.1154 10937514

[172] Bahar A Kashi Z Daneshpour E Akha O Ala S. Effects of cabergoline on blood glucose levels in type 2 diabetic patients: A double-blind controlled clinical trial. Med (Baltimore) (2016) 95(40):e4818. doi: 10.1097/MD.0000000000004818 PMC5059036 27749534

[173] Andersen M Glintborg D. Metabolic Syndrome in Hyperprolactinemia. Front Horm Res. (2018) 49:29–47. doi: 10.1159/000486000. 29894997

